# Lignin Stabilization and Carbohydrate Nature in H‐transfer Reductive Catalytic Fractionation: The Role of Solvent Fractionation of Lignin Oil in Structural Profiling**

**DOI:** 10.1002/cssc.202201875

**Published:** 2022-12-21

**Authors:** Raul Rinken, Dean Posthuma, Roberto Rinaldi

**Affiliations:** ^1^ Department of Chemical Engineering Imperial College London South Kensington Campus SW7 2AZ London UK

**Keywords:** biomass valorization, catalysis, fractionation, lignin-first, nickel

## Abstract

Reductive Catalytic Fractionation (RCF) of lignocellulosic materials produces lignin oil rich in monomer products and high‐quality cellulosic pulps. RCF lignin oil also contains lignin oligomers/polymers and hemicellulose‐derived carbohydrates. The variety of components makes lignin oil a complex matrix for analytical methods. As a result, the signals are often convoluted and overlapped, making detecting and quantifying key intermediates challenging. Therefore, to investigate the mechanisms underlining lignin stabilization and elucidate the structural features of carbohydrates occurring in the RCF lignin oil, fractionation methods reducing the RCF lignin oil complexity are required. This report examines the solvent fractionation of RCF lignin oil as a facile method for producing lignin oil fractions for advanced characterization. Solvent fractionation uses small volumes of environmentally benign solvents (methanol, acetone, and ethyl acetate) to produce multigram lignin fractions comprising products in different molecular weight ranges. This feature allows the determination of structural heterogeneity across the entire molecular weight distribution of the RCF lignin oil by high‐resolution HSQC NMR spectroscopy. This study provides detailed insight into the role of the hydrogenation catalyst (Raney Ni) in stabilizing lignin fragments and defining the structural features of hemicellulose‐derived carbohydrates in lignin oil obtained by the H‐transfer RCF process.

## Graphical abstract

**Simplicity is the ultimate sophistication**. β‐O‐4 linkages are found to undergo selective hydrodeoxygenation forming β‐O‐4 linkages bearing a methylene group at the C_α_ position. This process also stabilizes lignin fragments. In addition, carbohydrates in RCF lignin oil are found to be oligo(arabino)xylan alcohols, which are not covalently bonded to lignin.

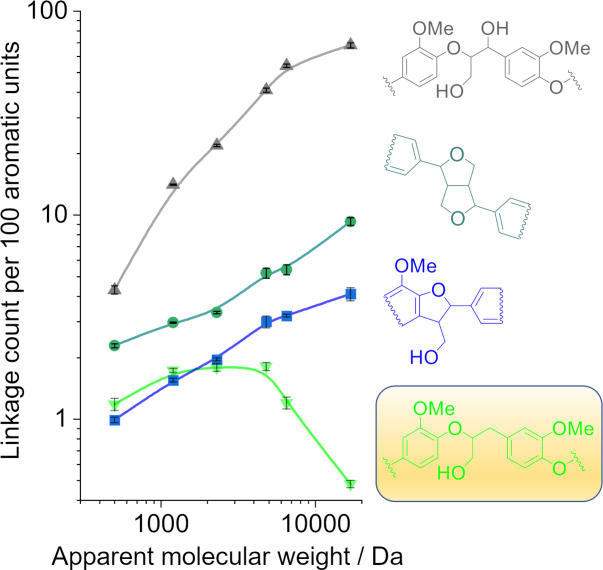

## Introduction

Reductive catalytic fractionation (RCF) is a promising lignin‐first approach for the biorefining of lignocellulosic materials.[^1^, ^2^, ^3^, ^4^, ^5^, ^6^, ^7^, ^8^] RCF relies on lignin stabilization by a hydrogenation catalyst in the presence of H_2_ or an H‐donor (e. g., 2‐propanol in H‐transfer RCF).[^9^, ^10^, ^11^, ^12^, ^13^, ^14^, ^15^, ^16^, ^17^, ^18^, ^19^, ^20^] Reductive processes stabilize lignin fragments released from lignocellulosic materials. As a result, the fate of lignin changes dramatically. Unlike organosolv processes, which isolate lignin polymers, RCF obtains lignin oil containing a substantial fraction of ‘monomer products’ (i. e., monoaromatic phenolics). They are produced at high individual yields as primary products.[^9^, ^10^, ^11^, ^12^, ^13^, ^14^, ^15^, ^16^, ^17^, ^18^, ^19^, ^20^] RCF also isolates high‐quality pulps from lignocellulosic materials.[^13^, ^21^, ^22^] Most of the research efforts put into the RCF process have focused on maximizing the yield of monomer products and controlling the degree of functionalization on the alkyl sidechain. Nonetheless, lignin oligomers/polymers and carbohydrate products from hemicellulose may make up to 40 % of the lignin oil composition. Heavy lignin fractions (>2000 Da) are often more significant in lignin oil obtained by H‐transfer RCF using Raney Ni and 2‐propanol/water mixtures under low‐severity conditions.[^9^, ^23^] In former literature, the H‐transfer RCF method was called Catalytic Upstream Biorefining (CUB).[^9^, ^15^, ^21^, ^24^, ^25^]

Taking the fast and substantial progress in RCF strategies into account, it is surprising that there is still very little information about the structural features of lignin oligomers/polymers and carbohydrates available in the current literature. In this respect, RCF lignin oil fractionation can potentially reduce the complexity of the RCF lignin oil matrix. Hence, it is expected to aid in deconvoluting complex data (e. g., from heteronuclear single quantum coherence nuclear magnetic resonance spectroscopy, HSQC NMR measurements), facilitating the elucidation of the structural features of lignin oligomers/polymers and carbohydrates. Moreover, obtaining lignin oil fractions in different molecular weight (MW) ranges is essential to determining the structural heterogeneity of lignin products across the entire molecular weight distribution of the RCF lignin oil. Such structural profiling is the key to answering pending research questions about whether lignin stabilization encompasses the entire population of lignin fragments or limits only to low‐MW fragments. Therefore, to accelerate the progress in RCF research, environmentally friendly and facile methods for fractionating RCF lignin oil are needed.

Solvent fractionation of technical lignins has extensively been investigated. Gigli and Crestini^[26]^ recently published a comprehensive review on the fractionation of industrial technical lignins. They outlined that 85 % of fractionation processes are based on solvent fractionation, while 15 % utilize membrane‐based technologies.[^27^, ^28^, ^29^, ^30^, ^31^, ^32^] Concerning solvent fractionation, Gosselink et al.^[33]^ invented a method for technical lignin fractionation that uses lignin powder packed with inert particles in a column. A series of increasing polarity solvents are used to extract soluble lignin fractions. Leitner group recently reported a fractionation method using expanded solvent systems (2‐methyltetrahydrofuran/CO_2_). By adjusting the pressure of CO_2_ in the expanded solvent system, the method was demonstrated to be highly selective in obtaining lignin fractions at various MW ranges.^[34]^

In RCF research, solvent fractionation of lignin oil has primarily been employed to isolate low‐MW lignin products. Sels group made significant progress characterizing lignin dimers and trimers, using ethyl acetate (EtOAc) and *n*‐heptane to work up lignin oil from pine wood.[^35^, ^36^] However, despite the reported use of solvent fractionation of lignin oil as a work‐up method, solvent fractionation still needs to be developed as a method applied to the structural profiling of RCF lignin oligomers/polymers.

Herein, we report the use of solvent fractionation of RCF lignin oil for its structural profiling. The RCF lignin oil was obtained from poplar wood in the presence of Raney Ni as the catalyst and 2‐propanol (2‐PrOH) as the H‐donor and the main component of the lignin‐extracting liquor (2‐PrOH : H_2_O, 70 : 30, *v/v*) at 200 °C for 3 h. We found that solvent fractionation using environmentally friendly solvents – methanol (MeOH), acetone, and EtOAc – produces RCF lignin oil fractions in different MW ranges. This outcome allowed us to address the structural heterogeneity of lignin stream and elucidate distinctive structural features imprinted by Raney Ni on lignin products and carbohydrates in the H‐transfer RCF process. In this report, the results and discussion are organized into three main parts. The first presents the molecular foundations and a phenomenological analysis of the solvent fractionation of RCF lignin oil. The second part thoroughly examines the fractions using high‐resolution HSQC NMR spectroscopy. It elucidates structural features created by Raney Ni on lignin fragments and carbohydrates in RCF lignin oil. The third part discusses the Raney Ni role in stabilizing lignin fragments in the H‐transfer RCF process.

## Results and Discussion

Our study began with a serendipitous finding. When attempting to dissolve the RCF lignin oil in anhydrous acetone, we obtained a copious pale beige precipitate and a yellow solution. This finding was somewhat surprising because acetone and its mixtures with polar solvents (e. g., alcohols and water) are well known to present excellent lignin‐extracting properties, recently explored in several Organosolv process variants.[^37^, ^38^, ^39^] Intrigued by the initial finding, we decided to characterize the precipitate by elemental analysis and HSQC NMR spectroscopy, as will be discussed in more detail in the following sections. In short, we found that the pale beige precipitate was not a result of any hypothetical reaction of RCF lignin oil and acetone. Instead, the precipitate was formed by the fractional dissolution of the RCF lignin oil. The process dissolved the monomer products and low‐MW oligomers in acetone. In contrast, mid‐ and high‐MW lignin fragments and carbohydrates were isolated from acetone as the pale beige precipitate.

### Molecular foundations of solvent fractionation

RCF lignin oil features a broad MW distribution of lignin products. To adjust the selectivity of the solvent fractionation of RCF lignin oil to obtain different cuts of low‐ or high‐MW lignin fragments, we rationally expanded the fractionation approach, including anhydrous EtOAc and anhydrous MeOH. We selected these solvents considering their Hansen solubility parameter (HSP) values and those of different types of lignins and lignin‐derived monophenols (Table 1).

**Table 1:** Hildebrand solubility parameter (*δ*) and HSP (*δ*_D_, *δ*_P_, *δ*_H_) for lignin, lignin‐derived monophenols, and selected solvents.

Solvent	*δ* [MPa^1/2^]	*δ*_D_ [MPa^1/2^]	*δ*_P_ [MPa^1/2^]	*δ*_H_ [MPa^1/2^]
Lignins[^41^, ^42^, ^43^]	24.6–31.0	16.7–21.9	13.7–18.4	11.7–16.9
Lignin‐derived monophenols[^44^, ^45^]	22.1–26.7	17.0–19.3	4.3–8.2	12.5–17.3
MeOH	29.6	15.1	12.3	22.3
Acetone	19.9	15.5	10.4	7.0
EtOAc	18.2	15.8	5.3	7.2
Water	47.8	15.5	16.0	42.3

**Scheme 1:**
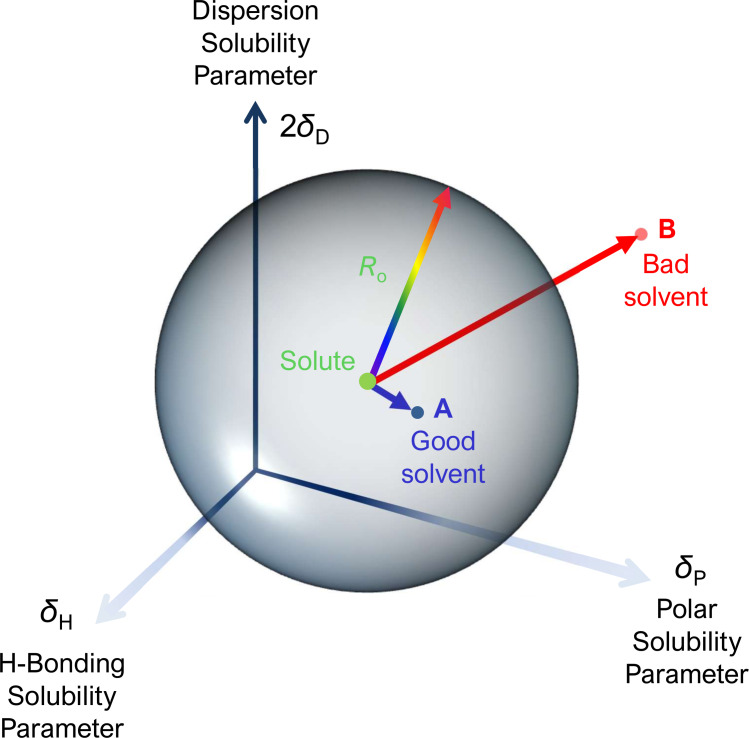
A sphere of radius *R*_o_ centered on the solute HSP values describes the HSP space in which dissolution is predicted to occur. The dissolution of a solute in a solvent **A** is expected when the relative energy difference (RSD), given by $\mathrm{RSD}=[4(\delta_{D}^{\mathrm{solute}}-\;\delta_{D}^{\mathrm{solvent}}{)}^{2}+\;(\delta_{P}^{\mathrm{solute}}-{\;\delta }_{P}^{\mathrm{solvent}}{)}^{2}+\;(\delta_{H}^{\mathrm{solute}}-{\;\delta }_{H}^{\mathrm{solvent}}{)}^{2}{]}^{0.5}$ , is smaller than the *R*_o_ value. No dissolution is expected in solvent **B** since RSD>*R*_o_.

HSP values are useful in the prediction of solubility. In the Hansen solubility theory, the Hildebrand parameter, *δ*, is mathematically decomposed into three parameters, namely, *δ*_D_ (for dispersion forces), *δ*_P_ (for polar interactions), and *δ*_H_ (for hydrogen bonding). Generally, a solute and a solvent sharing similar HSP values should be miscible and form a solution. This idea constitutes the foundation of the well‐known rule of “like dissolves like.” Quantitatively, the solubility prediction is made using a three‐dimensional representation of an HSP space contained in a sphere (Scheme 1). The solute HSP values define the sphere center coordinates. In this representation, a ‘good’ solvent has HSP coordinates within the sphere, whereas a ‘poor’ solvent presents its HSP coordinates beyond the sphere limit. Hence, the sphere radius (*R*_o_) indicates the maximum difference in HSP values tolerable for a “good” solute‐solvent interaction that leads to the dissolution of the solute in the solvent. The HSP and *R*_o_ values are usually determined by comparing the solute solubility in several solvents.[^40^, ^41^, ^42^]

For lignins, both HSP and *R*_o_ values are expected to be sensitive to the structural changes that occur in the lignin fragments during their extraction from lignocellulosic materials.^[71]^ Moreover, the MW distribution of the isolated lignin should also play a role in defining the *R*_o_ values. Technical lignins (e. g., Kraft lignins)[^41^, ^42^] and more ‘native‐like’ lignins (e. g., milled wood lignin)^[43]^ present HSP parameter values within 16.7–21.9 MPa^1/2^ for *δ*_D_, 13.7–18.4 MPa^1/2^ for *δ*_P_, and 11.7–16.9 MPa^1/2^ for *δ*_H_ (see Table S1 for an itemized list of lignins considered in the intervals). In addition, lignin dissolution is predicted within *R*_o_ values between 13.5 and 13.7 MPa^1/2^.^[41]^ Isolated lignins constitute a rather complex mixture of polymers, featuring non‐regular polymer chains in a wide MW range. In this manner, the HSP values for a lignin sample correspond to a weighted average of the individual set of HSP values for each lignin chain present in a sample. As a result, the dissolution extent of a lignin sample corresponds to fractionating a mixture of lignin polymers by matching the HSP values of a solvent or a solvent mixture with the HSP values for a particular lignin fraction in the sample. Therefore, when there is a match of HSP values, the fraction is dissolved and thus isolated from the remaining insoluble fraction.

Unlike technical lignins, which are *de facto* mixtures of degraded lignin polymers, RCF lignin oil consists of monomer products, stabilized lignin oligomers/polymers, and carbohydrates. Regarding the lignin fraction, by analyzing the HSP values for lignins and lignin‐derived monophenols, we found that they present similar values of *δ*_D_ and *δ*_H_ (see Table S2 for an itemized list of compounds included in our analysis). However, the *δ*_P_ values of lignin‐derived monophenols are lower than those of lignins. Hence, we reasoned that exploring the *δ*_P_ parameter could help select solvents to separate low‐MW lignin products from lignin oligomers/polymers in the RCF lignin oil. Owing to the different *δ*_P_ values, the solvents chosen for the fractionation of RCF lignin oil could lead to different cuts of low‐ or high‐MW lignin species as soluble or insoluble fractions, respectively. In this quest, water, which presents a high *δ*_P_ value (16.0 MPa^1/2^), can contribute to leveling the *δ*_P_ value of MeOH, acetone, or EtOAc to match the *δ*_P_ values found for lignins. In such a case, the dissolution extent of lignin oligomers/polymers from the RCF lignin oil would increase. Thus, anhydrous solvents and dried RCF lignin oil are needed for the result reproducibility of the solvent fractionation.

### Phenomenological analysis of solvent fractionation

**Figure 1:**
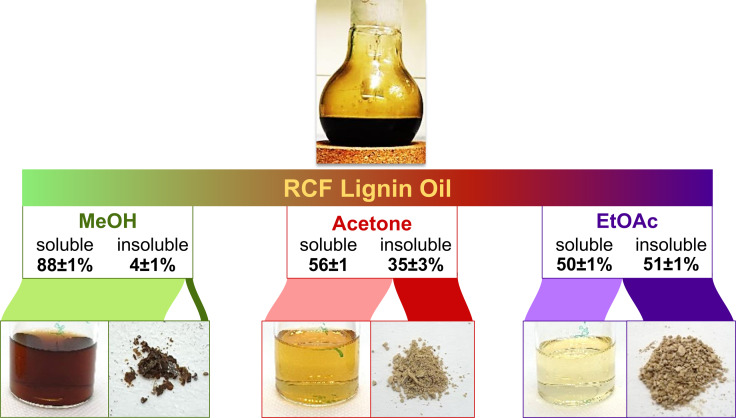
Initial observations of the solvent fractionation of RCF lignin oil in MeOH, acetone, or EtOAc. The pictures present the visual appearance of the soluble and insoluble fractions. The pictures from the soluble fractions were taken from the supernatant solution. The initial mass of RCF oil dispersed in each solvent was identical. Losses of volatile components (carried by solvent removal through rotary evaporation) are associated with an overall recovery below 100 %. In our experiments, the overall recovery was higher than 90 %.

Figure 1 summarizes the solvent fractionation results showing the visual appearance of the isolated fractions obtained by fractionating the RCF lignin oil using MeOH, acetone, or EtOAc. The RCF lignin oil presented the highest solubility in MeOH (88±1 % in MeOH vs. 56±1 % in acetone vs. 50±1 % in EtOAc). As a result, the RCF lignin oil dissolved in MeOH led to the darkest solution. After drying, the MeOH‐insoluble fraction was recovered as a hard, dark reddish‐brown solid. This insoluble fraction corresponded to 4±1 % of the RCF lignin oil in weight. The solvent fractionation of RCF lignin oil in acetone and EtOAc yielded yellow solutions; however, the acetone solution was darker. The acetone‐insoluble fraction represented 35±3 % of the RCF lignin oil in weight, while the EtOAc‐insoluble fraction was 51±1 %. Both acetone‐ and EtOAc‐insoluble fractions were recovered as pale beige‐colored soft solids. These solids are hygroscopic materials. Surprisingly, they can turn back to a very viscous oil when exposed to humidity.

**Figure 2:**
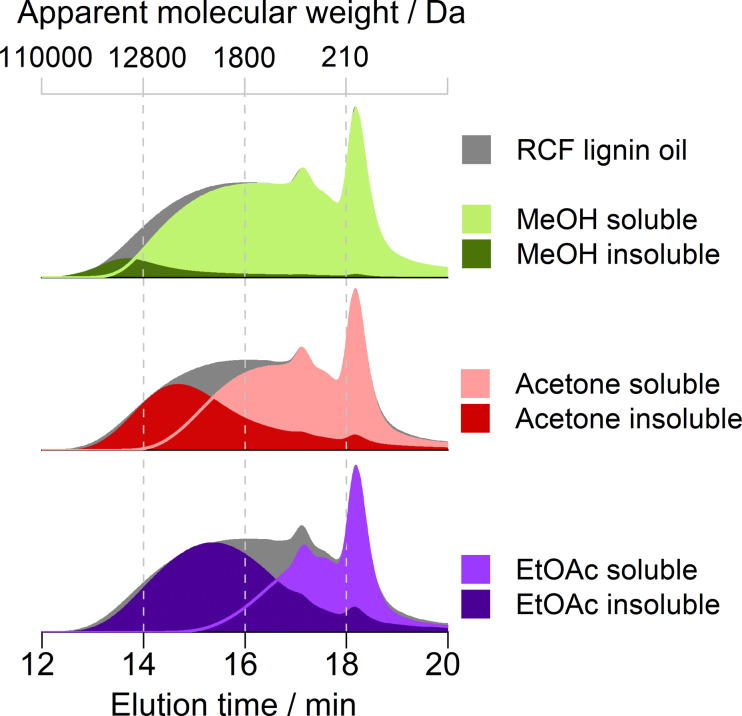
GPC traces for RCF lignin oil and its fractions insoluble or soluble in MeOH, acetone, or EtOAc. The traces were recorded at a wavelength of 270 nm.

The apparent MW distribution of the fractions was obtained by gel permeation chromatography (GPC) to examine the selectivity of the solvent fractionation (Figure 2). Table 2 summarizes the values for GPC trace onset and the apparent weight‐average molecular weight (*M_w_* not to confuse with MW). Solvent fractionation of RCF lignin oil produced a MeOH‐insoluble fraction showing an apparent *M_w_* value of 16900 Da and a chromatogram signal narrower than the RCF lignin oil's (Figure 2). GPC data shows that mid‐MW lignin fragments (from 1900 to 13600 Da) were less abundant in the acetone‐insoluble fraction than in the EtOAc counterpart. This observation implies that mid‐MW lignin fragments were more soluble in acetone than EtOAc. As a result, the acetone‐fraction insoluble presented an *M_w_* value higher than the EtOAc‐fraction insoluble (6500 Da vs. 4800 Da, Table 2). As determined by gas chromatography (GC), the insoluble fractions only contained trace amounts of monomer products (<0.1 wt % in total).

**Table 2:** Apparent values of *M_w_* the RCF lignin oil and its insoluble and soluble fractions in MeOH, acetone, and EtOAc.

Fraction	GPC trace onset [Da]	Apparent *M_w_* [Da]
RCF Lignin oil	36600	3400
MeOH‐insoluble	60700	16900
MeOH‐soluble	20500	2300
Acetone‐insoluble	32000	6500
Acetone‐soluble	8700	1200
EtOAc‐insoluble	29700	4800
EtOAc‐soluble	3000	500

**Figure 3:**
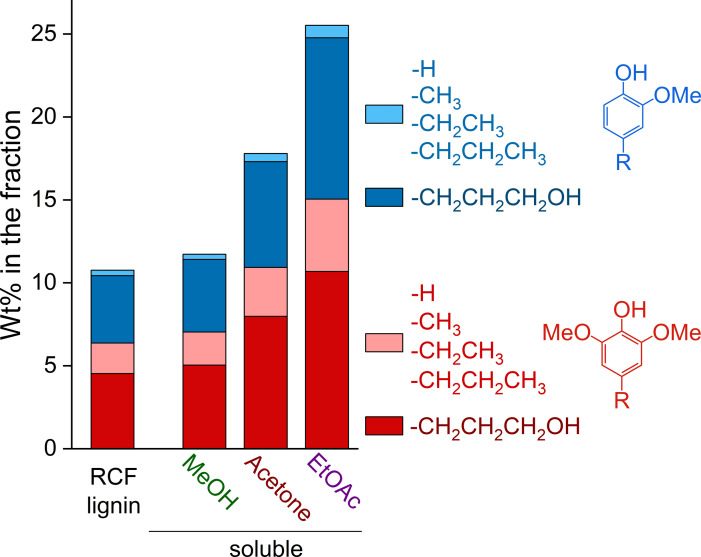
Content (wt %) of different lignin monomers in the RCF lignin oil and MeOH‐, acetone‐, and EtOAc‐soluble fractions as determined by GC‐FID/MS.

Regarding the soluble fractions of the RCF lignin oil, the selective removal of heavy species decreased the GPC trace onsets from 36600 Da (for the RCF lignin oil) to 20500 Da for the MeOH‐soluble fraction, 8700 Da for the acetone‐soluble fraction, and 3000 Da for the EtOAc‐soluble fraction (Figure 2). Hence, the apparent *M_w_* values decreased from 3400 Da (for the RCF lignin oil) to 2300 Da for the MeOH‐soluble fraction, 1200 Da for the acetone‐soluble fraction, and 500 Da for the EtOAc‐soluble fraction (Table 2). Moreover, the ability of acetone and, particularly, EtOAc to selectively dissolve low‐MW lignin species implies that the soluble fractions became proportionally enriched in monomer products. As shown in Figure 3, the content of monomer products progressively increased from 11 wt % in the RCF lignin oil to ultimately 26 wt % in the EtOAc‐soluble fraction.

The high selectivity towards low‐MW products found for the EtOAc fractionation of RCF lignin oil agrees well with the similarity of HSP between monomer products and EtOAc. In this instance, the *δ*_P_ parameter for EtOAc (5.3 MPa^1/2^) is particularly much closer to those of the monomer products (4.3–8.2 MPa^1/2^) than those reported for lignins (14.1–18.4 MPa^1/2^), as listed in Table 1. Furthermore, EtOAc also presents HSP much more distant from those reported for lignins. This feature makes EtOAc less suitable for dissolving mid‐MW lignin oligomers and polymers.

**Figure 4:**
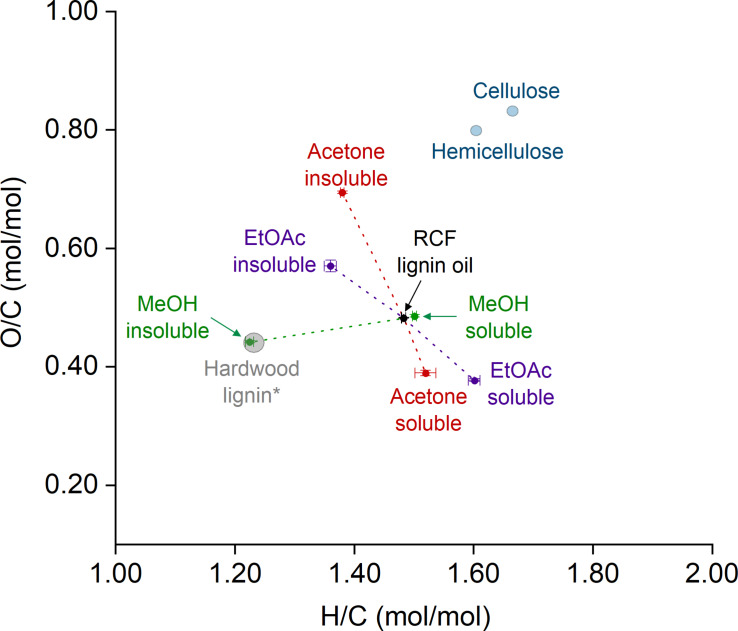
Van Krevelen diagram arranging the RCF lignin oil and its different fractions according to their H/C and O/C coordinates. The grey circle region is centered on the O/C and H/C hypothetical values for hardwood lignins (for details, see Figure S1). The blue circles present the regions for the H/C and O/C coordinates of cellulose and hemicellulose. The dashed lines guide the eye to the soluble and insoluble fractions obtained by fractionation in each solvent.

To evaluate the impact of solvent fractionation on the elemental composition of the fractions, we performed elemental analysis and arranged the H/C and O/C values of the fractions and RCF lignin oil in a Van Krevelen diagram (Figure 4). The insoluble fractions were distributed over the region within O/C from 1.22 to 1.38 and H/C from 0.44 to 0.69. Notably, the MeOH‐insoluble fraction showed statistically identical H/C and O/C values to those estimated for hypothetical hardwood lignin (actual vs. estimated value: H/C 1.22 vs. 1.25, O/C 0.44 vs. 0.44; for details, see the Supporting Information, Figure S1). Contrary to this, H/C and O/C values found for the acetone‐ and EtOAc‐insoluble fractions became more distant from the MeOH‐insoluble fraction coordinates. Their H/C and O/C values were shifted towards hemicellulose (H/C≈1.60 and O/C≈0.80) and cellulose (H/C≈1.67 and O/C≈0.83). This result indicated that both acetone‐ and EtOAc‐insoluble fractions contain a considerable amount of carbohydrates in their composition.^[24]^

Unsurprisingly, the RCF lignin oil and the MeOH‐soluble fraction presented similar H/C and O/C values, owing to the high solubility of RCF lignin oil in MeOH. In turn, the acetone‐ and EtOAc‐soluble fractions showed O/C values lower than those expected for lignin (0.38 vs. 0.44), with H/C values much higher than expected for lignin (1.52 and 1.60 vs. 1.22). In this instance, the high H/C can partly be attributed to the large share of HDO products from lignin. The low‐MW lignin products feature alkyl sidechains extensively hydrodeoxygenated (i. e., owing to the full removal of *O*‐functionalities on C_α_ and C_β_ and, to a much lesser degree, on the C_γ_ position). Furthermore, diols and polyols derived from hemicellulose also contributed to an increase in both H/C and O/C values.[^9^, ^46^, ^47^]

### HSQC NMR characterization of the RCF lignin oil fractions

**Figure 5:**
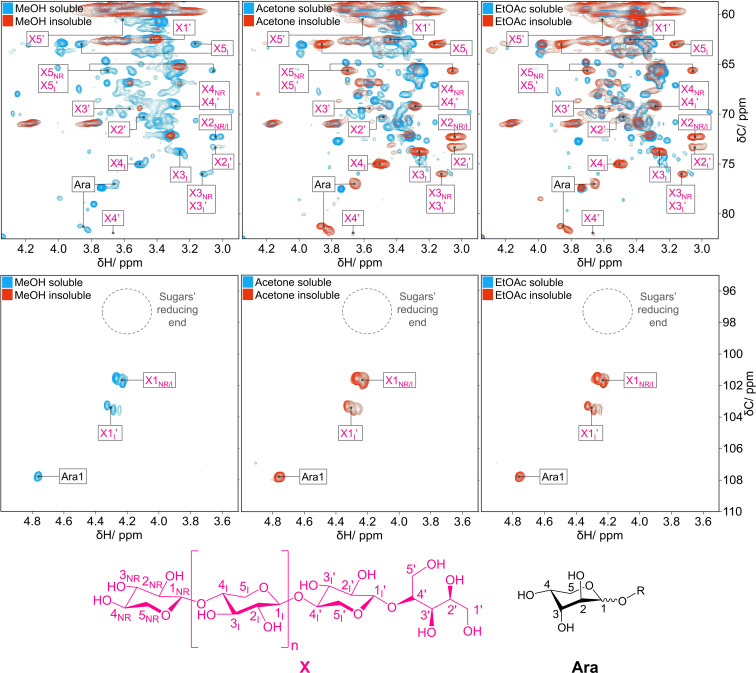
HSQC NMR spectra of lignin oil fractions highlighting the sugar signals in the oxygenated aliphatic region (top) and anomeric center region (bottom). For each solvent, the soluble fraction spectrum (in blue) is overlaid on the insoluble fraction spectrum (in red). The dotted circle region indicates the missing signals for the anomeric centers at the oligosaccharide reducing end. ‘X*#*_NR/I_’ and ‘Ara*#*’ identify the carbohydrates signals in the spectra; the numeral ‘*#’* stands for the carbon atom number labeling. ‘X_NR_’ and ‘X_I_’ indicate the ^13^C−^1^H correlation signals for non‐reducing xylopyranose and internal xylopyranose units, respectively. ‘Ara’ denotes the ^13^C−^1^H correlation signals for the anhydroarabinose unit. The oligosaccharide structure solely outlines key structural features in the backbone of the oligoxylan alcohols. Importantly, it does not intend to represent the actual structure of the oligo(arabino)xylan alcohols.

**Figure 6:**
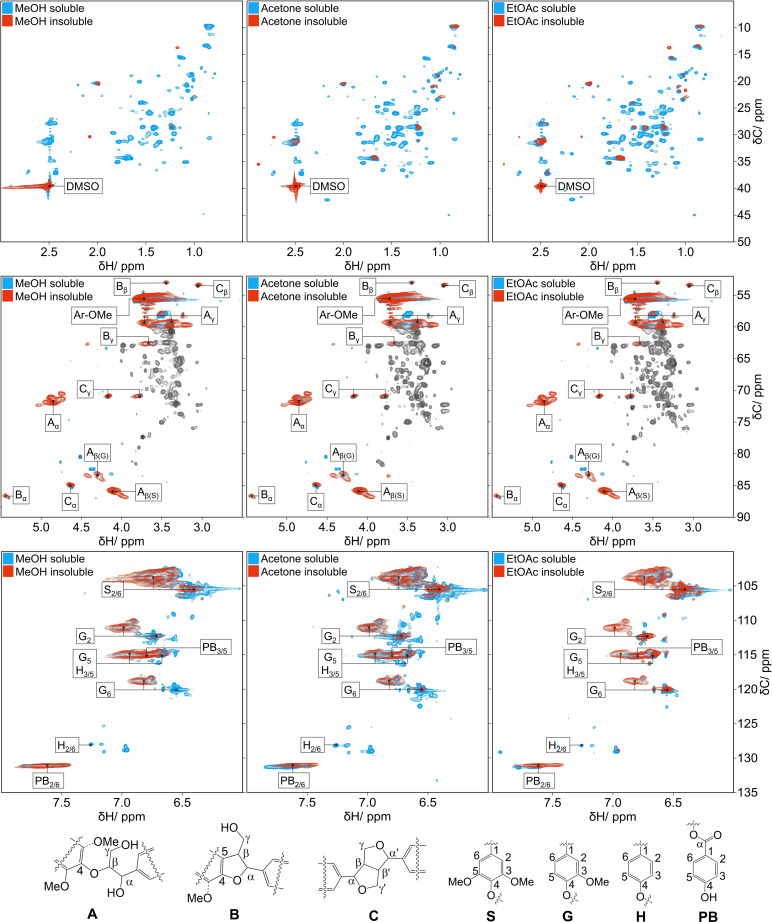
HSQC NMR spectra of lignin oil fractions zoomed on the aliphatic, oxygenated aliphatic, and aromatic regions. The soluble and insoluble fractions are superimposed to outline the difference between the two spectra. For clarity, we greyed out carbohydrate ^13^C−^1^H correlation signals to emphasize the signals for the canonical lignin linkages.

To address the structural features distinguishing the RCF lignin oil fractions, 2D HSQC NMR measurements of the RCF lignin oil (Figure S2) and fractions in [D_6_]DMSO were carried out. The analyses were performed on an 800 MHz NMR spectrometer equipped with a triple‐resonance cryoprobe. In the following sections, we compare the HSQC NMR spectra in selected regions. We present each soluble fraction spectrum (in *blue*) overlaid on the corresponding insoluble fraction spectrum (in *red*). For clarity, Figures 5 and 6 present the HSQC NMR spectra at a threshold aiding the identification of the main spectral features. In contrast, Figures S3–S8 display the individual HSQC spectra at a low threshold to highlight the less intense spectral features.

### Structural features of carbohydrates in RCF lignin oil

In the current RCF literature, little information is available on the chemical nature of the carbohydrate fraction in RCF lignin oil.[^3^, ^46^, ^47^] In this section, we detail the key structural features of the carbohydrate fraction. Figure 5 displays the HSQC spectra zoomed in the oxygenated aliphatic region (*δ*C/*δ*H=58–85/2.8–4.1 ppm) and anomeric center region (*δ*C/*δ*H=95–110/3.5–5.0 ppm). From Figure 5, it is clear that carbohydrate ^13^C−^1^H correlation signals constitute one of the main differences among the fractions. In the HSQC spectrum of the MeOH‐insoluble fraction, no ^13^C−^1^H correlation signals characteristic of carbohydrates were detected. This fraction presented ^13^C−^1^H correlation signals solely associated with lignin structural features.

The MeOH‐soluble fraction carried over carbohydrates together with lignin fragments. The carbohydrate signals are indicated on the HSQC NMR spectra by ‘X_*n*_’ (for xylopyranose units) and ‘Ara_*n*_’ (for arabinofuranose units), where *n* stands for the carbon atom numbering. Likewise, alongside the signals characteristic of lignin's structural features, the HSQC NMR spectra of the acetone‐ and EtOAc‐insoluble fractions presented the ^13^C−^1^H correlation signals close to those expected for (arabino)xylans, as will be discussed later in more detail. Despite the ^13^C−^1^H correlation signals for (arabino)xylans, no correlation signals were detected for the xylans’ reducing end terminal. This observation implies that the xylans converted into their corresponding alcohols by reductive processes catalyzed by Raney Ni. Even though this information is unprecedented in the RCF literature, the formation of oligo(arabino)xylan alcohols is plausible under reductive conditions.

HSQC NMR measurements indicated that acetone‐ and EtOAc‐insoluble fractions also contained mid‐ to high‐MW lignin fragments. We reasoned that, provided the carbohydrates were not covalently bonded to mid‐ to high‐MW lignin fragments, the carbohydrates could be extracted by hot water. To shed light on the connectivity between carbohydrates and lignin fragments, we washed the acetone‐insoluble fraction with hot water, a procedure much milder than the RCF process (200 °C for 3 h). Both soluble and insoluble fractions in water were collected, dried, and analyzed by the HSQC NMR technique. The HSQC NMR spectra (Figures S9 and S10) showed the water‐soluble products to be composed of carbohydrates with no trace of lignin fragments. In turn, the residue insoluble in hot water was a carbohydrate‐free lignin fraction. The carbohydrates presented identical spectral features to those found in the untreated acetone‐insoluble fraction. Therefore, we concluded that the carbohydrates were not covalently bonded to lignin fragments in the RCF lignin oil.

Analyzing the anomeric region in more detail revealed that the current HSQC NMR spectra were much less convoluted than those reported for the whole plant cell HSQC NMR spectra in pioneering work by Ralph group.[^48^, ^49^] In Figure 5, the anomeric center region displayed only two sets of ^13^C−^1^H correlation signals in addition to a single correlation signal at 107.81/4.76 ppm. The latter appears to be associated with the anomeric center of arabinose (Ara1) found in 4‐*O*‐methylglucuronoarabinoxylans.^[50]^ The first set of correlation signals (101.47/4.26 ppm; and 101.82/4.22 ppm) were associated with the ^13^C−^1^H pair on the C1 atom of non‐reducing (X_NR_) and internal xylopyranose units (X_I_) in the β‐(1→4)‐d‐xylopyranose backbone, as deduced from HSQC spectra of xylan^[49]^ and xylobiose (Figure S11). The second set of correlation signals (103.41/4.32 ppm, 103.58/4.25 ppm, 103.58/4.28 ppm) overlapped with the reported values for the ^13^C−^1^H pair on the C1 atom of the anomeric correlation signal from β‐(1→4)‐d‐Glc*p* in the cellulose's non‐reducing‐end terminal.^[49]^ Notwithstanding, considering the HSQC NMR spectra of cellobiose and cellobiitol (Figure S12), no other correlation signals corresponding to the ^13^C−^1^H pairs of glucopyranose were found in the oxygenated aliphatic region. This observation confirmed that no glucopyranose units were present in the oligosaccharides of the RCF lignin oil fractions.

Motivated by these observations, we synthesized xylobiitol as a model to verify whether the second set of correlation signals is related to the oligoxylan alcohol backbone. Xylobiitol was obtained from xylobiose by reduction with NaBH_4_ under ambient conditions.^[51]^ Comparing the spectra from Figure 5 against HSQC NMR spectra of xylobiose and xylobiitol (Figure S9) confirmed that the second set of peaks unequivocally corresponds to the ^13^C−^1^H pair on the C1_I′_ of the penultimate xylopyranose unit of the oligoxylan alcohols. This information also allowed us to estimate the average degree of polymerization of the β‐(1→4)‐d‐xylopyranose backbone. From the volume integrals of the anomeric center ^13^C−^1^H correlation signals, the average composition of the β‐(1→4)‐d‐xylopyranose backbone in the RCF lignin oil was estimated at *ca*. four xylopyranose units with an average occurrence of 0.5 arabinofuranose unit relative to the total count of xylopyranose units (see Supporting Information).

A key feature of Poplar hemicellulose is the acetylation of 4‐O‐methylglucuronoxylan on the C2 and C3 positions of xylopyranose units. From previous HSQC NMR studies for whole plant cell samples dissolved in the [D_6_]DMSO/[D_5_]pyridine (4 : 1, v/v), the correlation signals for the ^13^C−^1^H pair at C2 (ca. 73.5/4.64 ppm in 2‐*O*‐Ac‐β‐d‐Xyl*p*) and ^13^C−^1^H pair at C3 (ca. 75.0/4.94 ppm in 3‐*O*‐Ac‐β‐d‐Xyl*p*) were demonstrated to serve as a clear structural signature for the presence of acetylated xylans.^[50]^ Although acetyl groups were found in our spectra (20.51/1.99 ppm), the absence of correlation signals for 2/3‐*O*‐Ac‐β‐d‐Xyl*p* indicated no acetylation of the oligo(arabino)xylan alcohols. In fact, the deacetylation of xylans by hydrolysis in the liquor solvents (2‐PrOH/H_2_O, 70 : 30, v/v) was observed under the RCF conditions.^[46]^

### Structural features of lignin fragments

To investigate the main structural features of lignin fragments in the fractions, we examined the overlaid HSQC spectra from the soluble and insoluble fractions in: (i) C−H aliphatic region (*δ*H/*δ*C=0.5–3.0/5–50 ppm); (ii) oxygenated aliphatic region (*δ*H/*δ*C=2.5–5.5/50–90 ppm); and (iii) aromatic region (*δ*H/*δ*C=6.0–8.0 ppm). In Figure 6, the comparison between HSQC NMR spectra of soluble (in blue) and insoluble fractions (in red) revealed marked differences in the C−H aliphatic region. The spectra of the soluble fractions presented a very complex pattern of ^13^C−^1^H correlation signals due to the variety of monomer and oligomer products (with deoxygenated alkyl sidechains) occurring in the soluble fractions. In turn, the HSQC NMR spectra of the insoluble fractions displayed only a few ^13^C−^1^H correlation signals. This observation demonstrated that HDO processes on the alkyl sidechains were much less extensive on the mid‐ and high‐MW lignin fragments compared to the low‐MW lignin products.

In the HSQC NMR spectra, both insoluble and soluble fractions presented the ^13^C−^1^H cross signals characteristic of the canonical linkages found in native lignins (A: β‐O‐4 linkages, and B: phenylcoumaran and C: resinol interunit bonding motifs). As expected, the ^13^C−^1^H pair for guaiacyl (G) and syringyl (S) units were the prevalent signals in the aromatic region. As secondary lignin features, we detected the ^13^C−^1^H pairs for PB_2/6_ and PB_3/5_ from the *p*‐hydroxybenzoate (PB) groups. They decorate the main backbone of lignin polymer as pendant groups.^[52]^ The apparent abundance of PB pendant groups relative to combined G‐ and S‐units decreased from 7.8±0.3 % (16900 Da, MeOH‐insoluble fraction) to 1.9±0.1 % (500 Da, EtOAc‐soluble fraction; Figure S13). Surprisingly, ^13^C−^1^H cross signals characteristic of H_2/6_ and H_3/5_ from coumaryl (H) units were only present in the low‐MW soluble fractions (ca. 0.3 % relative to combined H‐, G‐, and S‐units). Raney Ni is active in the demethoxylation of guaiacols under H‐transfer conditions.[^53^, ^54^, ^55^] Considering this information, it seems plausible that the demethoxylation of G‐type products might account for at least part of the minor amount of H‐type products found in the low‐MW fractions of RCF lignin oil.

**Figure 7:**
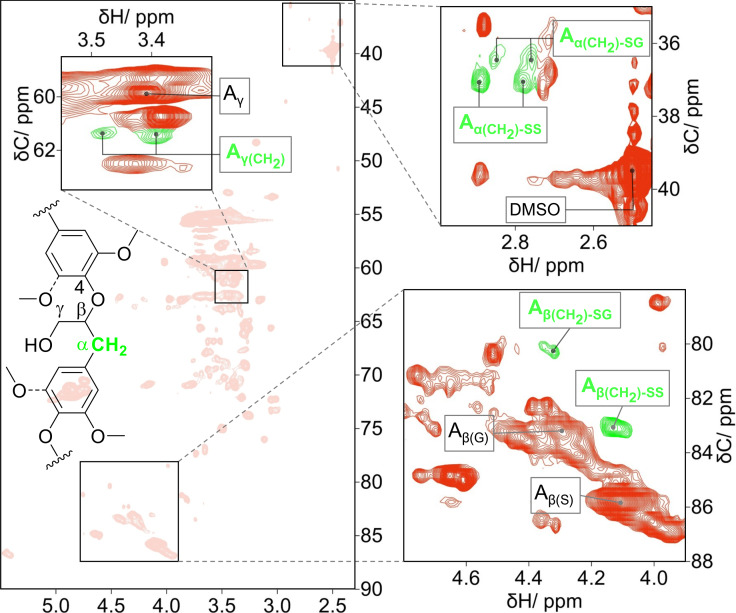
HSQC NMR spectrum of the EtOAc‐insoluble fraction dissolved in [D_6_]DMSO. The insets zoomed spectral regions are presented at a threshold lower than that chosen for clarity in Figures 5 and 6. The ^13^C−^1^H correlation signals attributed to the reduced β‐O‐4 linkages are highlighted in green.

A careful analysis of the ^13^C−^1^H correlation signals in a low threshold also revealed the presence of a non‐native lignin linkage created by the reductive processes on the β‐O‐4 interunit bonding motif. The non‐native linkage corresponds to a reduced β‐O‐4 linkage presenting a methylene group at the C_α_ position. In Figure 7, the ^13^C−^1^H correlation signals attributed to the reduced β‐O‐4 linkages are designated by A_x(CH2)_, where ‘x’ indicates the α, β, or γ position in the (reduced) β‐O‐4 linkage. The assignments agree well with those reported for β‐O‐4 lignin models and lignans presenting a methylene group at the C_α_ position.[^56^, ^57^, ^58^, ^59^] In contrast, such a feature could not be found in the reported HSQC NMR spectra of lignin oils obtained from RCF in the presence of a Ni/Al_2_O_3_ catalyst under H_2_ pressure.^[60]^ Notably, the absence of these ^13^C−^1^H correlation signals might result from a low signal‐to‐noise ratio in the HSQC NMR measurements of unfractionated RCF lignin oils or due to the choice of a high threshold for a ‘clearer’ presentation of the spectral data of RCF lignin oils, thus not acknowledging less intense signals in the HSQC NMR spectra. However, the formation of reduced β‐O‐4 linkages observes the chemoselectivity pattern found for HDO of model compounds containing *O*‐functionalities at the benzylic position (e. g., alcohol, aldehyde, ether, or a ketone group) when performed in the presence of Raney Ni and using 2‐propanol as the H‐donor.[^53^, ^54^]

The signals attributed to A_α(CH2)_ were 37.06/2.90 ppm and 37.06/2.78 ppm for the linkage formed between two syringyl (S) units, A_α(CH2)‐SS_. In addition, less intense signals at 36.53/2.86 and 36.53/2.78 ppm were assigned to the reduced β‐O‐4 linkage between a syringyl (S) and an etherified guaiacyl (G) unit, A_α(CH2)‐SG_. In turn, the ^13^C−^1^H correlation signals for A_β(CH2)‐SS_ and A_β(CH2)‐SG_ were detected at 83.32/4.10 ppm and 80.56/4.30 ppm, respectively. The correlation signals for A_γ(CH2)_ were expected in a region convoluted by several overlapping signals. Notably, the signals could be identified at 61.34/3.48 and 61.34/3.39 ppm. The chemical shifts agree well with those reported for A_γ(CH2)‐SS_ occurring in lignin models and isolated lignans.[^56^, ^57^, ^58^, ^59^] The integral ratio A_γ(CH2)_‐to‐(A_β(CH2)‐SS_+A_β(CH2)‐SG_) was equal to 2. Despite the semiquantitative nature of the HSQC NMR data, this observation suggests that the correlation signals for A_γ(CH2)‐SS_ and A_γ(CH2)‐SG_ were most likely overlapped in Figure 7.

**Figure 8:**
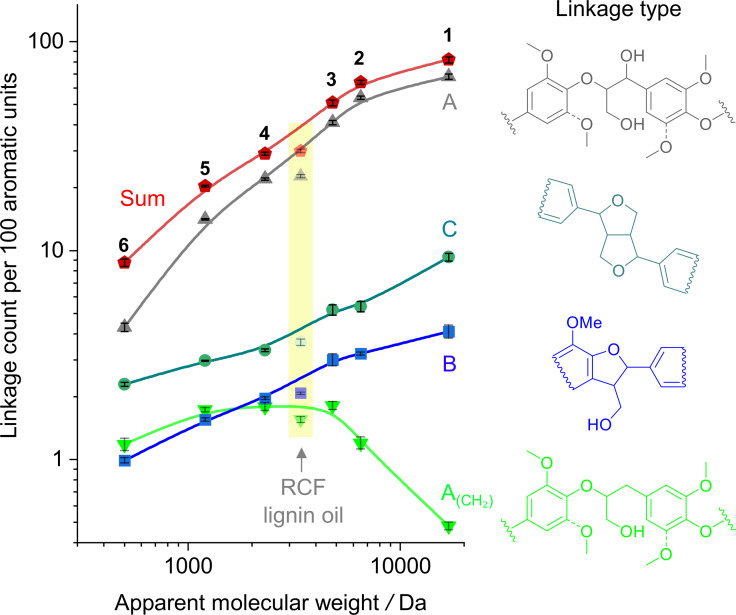
Distribution of estimated linkage counts per 100 aromatic units in the fractions relative to their apparent *M_w_* value. The axes present the values on logarithmic scales for clarity. The inset in yellow indicates the linkage count values for the RCF lignin oil relative to its apparent *M_w_* value. The numerals identify the fractions: 1. MeOH‐insoluble; 2. acetone‐insoluble; 3. EtOAc‐insoluble; 4. MeOH‐soluble; 5. acetone‐soluble; 6. EtOAc‐soluble.

To examine the structural heterogeneity of RCF lignin oil, we estimated the linkage counts (A, A_(CH2)_, B and C) per 100 aromatic units using the HSQC NMR spectral data (see Supporting Information). Figure 8 shows a diagram correlating the linkage counts per 100 aromatic units and the apparent *M_w_* value of the fractions. Both axes in Figure 8 were presented on a logarithmic scale to facilitate the data visualization. The linkage occurrence determined for RCF lignin oil was shifted to values lower than those expected from the trend lines at the apparent *M_w_* value of the RCF lignin oil. This shift was expected due to the high polydispersity of RCF lignin oil, which contained a significant fraction of monomer products. Amongst the fractions, the MeOH‐insoluble fraction presented the highest total linkage count per 100 aromatic units (82±2), followed by the acetone‐ and EtOAc‐ insoluble fractions (64±2 and 51±2, respectively). In turn, MeOH‐, acetone‐, and EtOAc‐soluble fractions showed total linkage counts (29±1, 20±1, and 9±1, respectively) lower than the insoluble fractions.

It is clear from Figure 8 that the β‐O‐4 linkages were the most prevalent ones in the fractions, followed by resinol and phenylcoumaran bonding motifs. The individual linkage counts per 100 aromatic units decreased for all canonical lignin linkages with the reduction in MW values. In the case of β‐O‐4 linkages, the decrease in linkage count is directly related to lignin depolymerization. In the case of phenylcoumaran and resinol interunit bonding motifs, the hydrogenolysis of C−O bonds is responsible for decreasing the linkage count. However, no depolymerization occurs since the aromatic units remain connected by an alkyl chain linker.[^35^, ^36^] Interestingly, the reduced β‐O‐4 linkages present a different trend from the canonical lignin linkages. In this instance, there was an increase in the linkage count (from 0.48±0.02 to ca. 1.81±0.08 per 100 aromatic units) with a decrease in apparent *M_w_* values from 16900 Da to 4800 Da. At this point, the trend presented a plateau lasting until 1200 Da (at about 1.8 linkage count per 100 aromatic units), when a final decrease followed it to 1.18±0.08 linkage count per 100 aromatic units at 500 Da.

The relative abundance of syringyl (S) and guaiacyl (G) units in hardwood lignin constitutes a key parameter defining the portfolio of lignin‐derived products obtained from RCF. Moreover, Beckham and Román‐Leshkov research groups demonstrated that the S/G ratio does not govern monomer yields.^[61]^ They proposed that the distribution of interunit bonding motifs containing C−O or C−C bonds is defined by the lignin monomer transport during lignin biosynthesis. Several studies indicated that the S/G ratio in the monomer products increased with the RCF process duration.[^61^, ^62^] At the initial stages, the more extensive extraction of G‐type lignin was proposed to take place, being attributed to uneven S−G lignin distribution in cell walls, with G‐type lignin domains being more accessible than the S‐type counterparts.[^61^, ^62^, ^63^]

**Figure 9:**
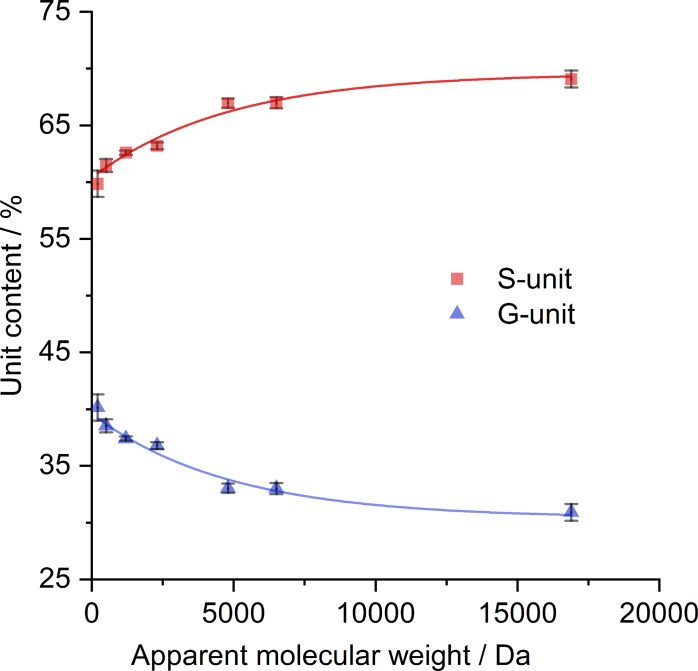
Correlation between the relative abundance of S‐ and G‐type units in the lignin fractions and their apparent *M_w_* value. The first point (ca. 200 Da) corresponds to the S‐ and G‐type volatile products determined by GC‐FID.

To examine the product distribution in terms of S‐ and G‐unit content in different fractions of RCF lignin oil, Figure 9 correlates the relative abundance of S‐ and G‐units against the apparent *M_w_* values of the fractions and reveals a key feature. The occurrence of S‐type units in the products gradually increased with molecular weight. Consequently, the products found an opposite trend in the occurrence of G‐type units. From GC data, S‐ and G‐type products corresponded to 60±1 % and 40±1 % of monomer products in the RCF lignin oil, respectively. The HSQC NMR spectral data indicated an increase in S‐unit abundance from 61.5±0.6 % to 69.1±0.8 %, while the G‐unit abundance decreased from 38.5±0.6 % to 30.9±0.8 % with the increase in molecular weight.

Previous reports[^61^, ^62^] provided convincing evidence indicating that the lignin fragments rich in G‐unit were released at the early phase of RCF (<1 h). Considering this, the lignin fragments rich in G‐units will invariably dwell longer in the liquor when the RCF process is performed in a batch reactor. This fact also implies that lignin fragments rich in G‐units have more time to be converted into low‐MW products. Our data also confirmed this working hypothesis. Low‐MW lignin products (200–1000 Da) were enriched in G‐units compared to mid‐ and high‐MW lignin products (Figure 9). In contrast, the slow release of lignin fragments rich in S‐units reduced their dwell time in the liquor. Consequently, the extent of conversion of S‐lignin fragments into low‐MW products is expected to be lesser compared to G‐lignin fragments. As a result, mid‐ to high‐MW lignin fragments became enriched in S‐units. Altogether, it is apparent from Figure 9 that the “history” of the lignin‐releasing processes and conversion was preserved in the RCF lignin oil composition.

### Insights into the stabilization of lignin

Stabilizing lignin fragments constitutes the essential feature of the lignin‐first biorefining concept. In RCF, the lignin fragments are stabilized against electrophilic aromatic substitution by selective hydrodeoxygenation (HDO) of Hibbert ketones. In other lignin‐first concepts, the stabilization of lignin is achieved by using protection or capping agents (e. g., formaldehyde or other aldehydes,[^64^, ^65^] primary alcohols,[^66^, ^67^] and diols[^68^, ^69^]). Stabilizing lignin fragments inhibits lignin condensation, which otherwise would degrade native lignin structures due to the formation of random non‐native C−C bonding motifs among the lignin fragments. In this context, it is now widely recognized that lignin condensation forms recalcitrant lignin‐derived polymers with a low potential for valorization.[^1^, ^7^]

Despite the current consensus on the importance of the stabilization of lignin fragments for a positive outcome in RCF,[^3^, ^7^] little is known about whether stabilization encompasses all lignin species in the liquor or limits to low‐MW lignin fragments. In this respect, the Dyson group reported an elegant study on the catalyst role in the RCF process.^[70]^ They utilized a designed catalyst comprising Rh nanoparticles embedded in the interior of hollow porous carbon nanospheres in the RCF of birchwood under H_2_ pressure. They found that porous carbon nanospheres could sieve reactive lignin monomer intermediates from polymers in the liquor. Moreover, supported by additional evidence obtained from model reactions using polystyrene or toluene as the substrate, they proposed that the stabilization via reductive processes occurs primarily on monomer products.^[70]^

**Figure 10:**
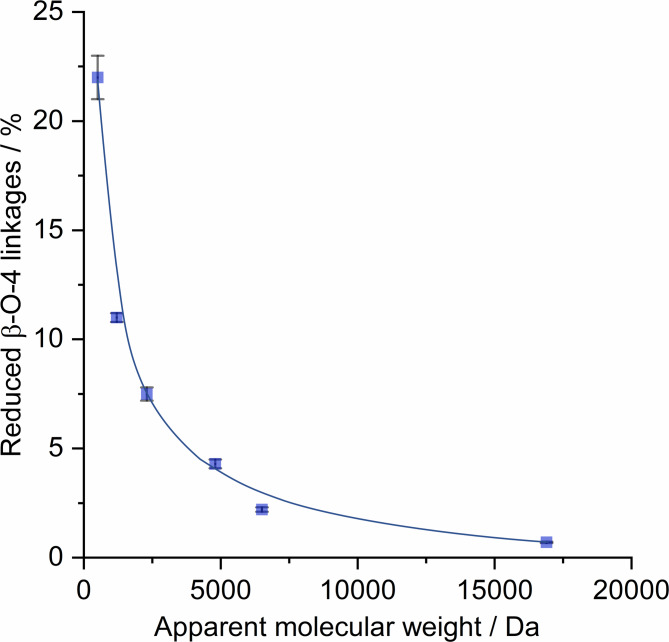
Correlation between the population of reduced β‐O‐4 linkages with the apparent value of *M_w_* of the fractions isolated by solvent fractionation of the RCF lignin oil.

**Scheme 2:**
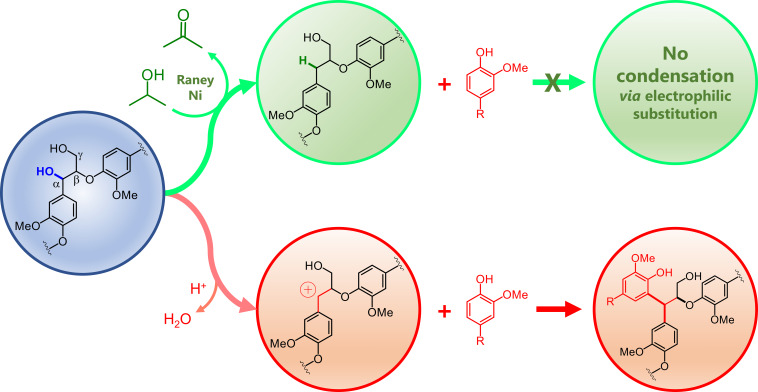
Route proposed for stabilization of lignin fragments through selective HDO of the C_α_ site. This reaction converts the C_α_−OH group into a methylene group. This impedes electrophilic aromatic substitution, thus suppressing lignin condensation.

In the current study, we found lignin fragments containing reduced β‐O‐4 linkages formed via selective HDO of the C_α_ position of native β‐O‐4 linkages in the entire population of lignin fragments. In fact, the population of reduced β‐O‐4 linkages exponentially decreased from 21 % to 0.7 % with the increase in apparent *M_w_* values from 500 Da to 16900 Da (Figure 10). As presented in Scheme 2, the presence of a CH_2_ group at the C_α_ position eliminates the possibility of forming a benzylic carbocation, effectively impeding lignin condensation at the reduced β‐O‐4 bonding motifs.

## Conclusions

We demonstrated the fractionation of RCF lignin oil using environmentally friendly solvents (acetone, EtOAc, methanol) to render fractions in different molecular weight ranges. This feature allowed us to determine the structural heterogeneity of lignin products across the entire molecular weight distribution of the RCF lignin oil. The detailed characterization of the fractions by HSQC NMR measurements demonstrated the formation of reduced β‐O‐4 linkages presenting a methylene group at the C_α_ position. It is generally accepted that the stabilization processes primarily occur on lignin monomer intermediates. Although this claim holds, our findings indicate that, through the formation of reduced β‐O‐4 linkages, an additional route for lignin stabilization is established. This process occurs to varying degrees in the entire population of lignin fragments.

Concerning the chemical nature of the carbohydrates in the RCF lignin oil, the carbohydrate fraction is composed of oligo(arabino)xylan alcohols. The carbohydrates were not chemically connected to lignin fragments. Therefore, they could easily be removed from the fractions by hot water extraction with no apparent degradation.

In the broad context, the current findings have far‐reaching implications for lignin valorization. Mirroring previous works by Argyropoulos group,[^72^, ^73^] demonstrating fractional precipitation as another effective solvent fractionation strategy for obtaining consistently homogeneous lignin streams from softwood Kraft lignins, the solvent fractionation of RCF lignin oil could also hold potential for commercial applications. In such an approach, the valorization of lignin is defined by the lignin stream fraction that provides the best properties for desired applications (e. g., to produce advanced materials).[^74^, ^75^, ^76^] In this respect, the stabilization imparted by reductive processes preserves the structure of high‐MW lignin in a more native‐like architectural state. For instance, the heaviest fraction (MeOH‐insoluble fraction, *M_w_* 16900 Da) presented an estimated total linkage count of 82±2 per 100 aromatic units. Consequently, heterogeneous catalysis can be crucial in generating mid‐ and high‐MW lignin products with controllable structural properties. Such control has thus far been only possible through the rational bioengineering of lignin in planta.^[1]^ However, upon lignin extraction, such precious structural features inserted by bioengineering are often lost. Therefore, the evolution of the RCF concept toward the production of lignin polymer products of controllable structural properties presents itself as an attractive ‘new’ option in the lignin‐first concept.

## Experimental Section

### Materials

Raney Ni (Raney Ni® 2800 slurry in H_2_O) was purchased from Sigma‐Aldrich. Poplar chips were obtained from premium animal bedding (Rettenmaier & Soehne). All solvents (HPLC grade, VWR) utilized as purchased. [D_6_]DMSO was purchased from Sigma‐Aldrich.

### H‐transfer RCF of poplar wood

Poplar wood chips (704 g) and Raney Ni (331 g, wet) were suspended in a 2‐PrOH/H_2_O mixture (4140 mL, 70 : 30 v : v) in a 9‐L) stainless steel batch reactor (Parr Instruments & Co, model number: 4552). The suspension was stirred at 75 rpm for 30 min at room temperature. Then, the reactor was heated to 200 °C for 1 h (3 °C/min) under mechanical stirring (75 rpm). The H‐transfer RCF process proceeded under autogenic pressure for 3 h. In sequence, the mixture was allowed to cool down to room temperature. The liquor was separated from the solids (Raney Ni in conjunction with the holocellulosic pulp) by filtration through a glass fiber filter (GF6, Whatman). The lignin oil stream was isolated from the liquor by removing the solvent mixture using a rotary evaporator at 45 °C under reduced pressure and further dried in a vacuum oven at 40 °C for 24 h.

### Solvent fractionation of RCF lignin oil

Anhydrous solvent (acetone, EtOAc, or methanol, 10 mL) was added to 0.4000 g lignin oil and sonicated for 30 min. The mixture was centrifuged at 5000 RCF for 10 min, and the supernatant was separated. The solid was redispersed in the solvent (10 mL), sonicated for 30 min, and centrifuged at 5000 RCF for 10 min. This procedure was repeated one more time. Next, the supernatants were combined, and solvent removed on a rotary evaporator at 40 °C. The isolated fractions were dried under reduced pressure (50 mbar) in a vacuum oven at 40 °C for 24 h. The mass of both fractions was recorded. The fractionation was repeated up to 5 times for each solvent, and the deviation is reported as the standard deviation for the repeats.

### Extraction of carbohydrates with hot water

To extract carbohydrates from the acetone‐insoluble fraction, the acetone‐insoluble fraction was suspended in hot distilled water (80 °C, 10 mL) and sonicated for 10 min. The mixture was centrifuged at 5000 RCF for 10 min, and the supernatant was separated. The solid was redispersed in the hot water (10 mL), sonicated for 30 min, and centrifuged at 5000 RCF for 10 min. This procedure was repeated one more time. The supernatants were combined and reduced on a rotary evaporator at 50 °C. The isolated fractions were dried under reduced pressure (50 mbar) in a vacuum oven at 40 °C for 24 h.

### Elemental analysis

Elemental analysis was performed on an Elementar VarioMICRO Cube CHNS/O elemental analyzer. The sample amount was 1.5–2.5 mg sample. Each analysis was repeated at least three times. The oxygen content in the sample was determined by subtraction. The deviation was calculated as standard deviation relative to H/C and O/C mole fractions.

### Gel‐permeation chromatography (GPC) measurements

The samples (10 mg) were dissolved in the mobile phase (1 mL, 0.1 wt % LiBr/DMF). The samples were analyzed on a Shimadzu Prominence HPLC equipped with an autosampler (SIL‐20A HT), oven (CTO‐20A), PDA (SPD‐M20A), and RI detector (RID‐20A). The separation was performed on a set of M and L PolarGel columns (300 mm×7.8 mm, Agilent) operating at 60 °C with a mobile phase flow of 1 mL/min. Apparent molecular weight calibration was performed against polystyrene standards (Agilent S‐L‐10, S‐M‐10) dissolved in the mobile phase. The chromatograms were analyzed using the Shimadzu GPC software suite (version 5.98). The PDA response was analyzed at the wavelength of 270 nm. Note: When applied to product mixtures obtained from lignin, quantitative information regarding the content of species cannot be retrieved from a PDA or RI detector, as the detector response is not universal. Despite this limitation, the GPC technique coupled with UV‐Vis spectroscopy provides valuable information allowing for a comparison of the apparent MW distribution.

### Gas chromatography analysis

An aliquot of each sample (50.0 mg) was dissolved in MeOH containing internal standard *n*‐decane at 1 mg/mL. The sample was filtered (membrane filter 0.45 μm). The sample solutions were analyzed by GC‐MS/FID 2010 Plus (Shimadzu) equipped with a capillary column DB‐1MS (30 m, 0.25 mm ID, df 0.25 μm). The following oven temperature program was used: an isothermal step for 5 min at 40 °C, then the temperature was ramped up at 5.2 °C min^−1^ to 320 °C and kept for 5 min. The products were identified by searching the mass spectra with the mass spectral libraries NIST 08, NIST 08s, and Wiley 9. Quantification of selected components was performed by using the flame ionization detector (FID) response and authentic product standards.

### HSQC NMR measurements

Samples were prepared by dissolving a sample (50 mg) in [D_6_]DMSO (1 mL). The samples were analyzed with an 800 MHz Bruker spectrometer equipped with inverse triple resonance cryoprobe with ATM module (5 mm ^1^H/^13^C/^15^N/D Z‐GRD). All measurements were performed at 25 °C. A matrix with 1024×2048 points was collected over four scans. Bruker *hsqcetgpsi* pulse program was used. All data were processed using MestreNova software (version 14.3.0). The considerations on HSQC data analysis and quantification are given in Supporting Information.

### Synthesis of xylobiitol

Xylobiose (0.7 mmol) was dissolved in deionized H_2_O (2.5 mL) in a 10 mL round‐bottomed flask. NaBH_4_ (1.5 mmol) was dispersed in deionized H_2_O (0.8 mL) for 1 min and added to the xylobiose solution right after. The reaction mixture was stirred (600 rpm) at room temperature for 1 h. Pre‐washed Amberlite IR120(H) resin was added to the reaction mixture until the gas separation stopped (2 mL in total), and the mixture was stirred for an additional 10 min. A 3‐cm column was packed on the P3 glass filter with fresh resin dispersed in water (10 mL), and the reaction mixture with the added resin was poured into the column. After gravitational flow had removed most of the water, the resin was washed three times with deionized H_2_O (20 mL). The filtrate was reduced to dryness on a rotary evaporator (at 40 °C under 30 mbar). MeOH (5 mL) was added to the residue, shaken for 10 min, and reduced to dryness on a rotary evaporator (at 40 °C under 200 mbar). This process was repeated two more times. The remaining residue was placed in a vacuum oven for 24 h (at 40 °C under 50 mbar). The crude product was further purified by recrystallization. MeOH (8 mL) was added to the solid, boiled to half volume, slowly cooled to room temperature, and further cooled down in an ice bath. The remaining solvent was removed by decantation. The final product was dried in a vacuum oven under 50 mbar at 40 °C for 24 h, resulting in a white solid (63 % yield). The product was characterized by 2D HSQC NMR in [D_6_]DMSO, indicating no impurities (Figure S12). The ^13^C−^1^H correlation signals assignment of the product is given in Table S4.

### Synthesis of cellobiitol

Cellobiose (2.9 mmol) was dissolved in deionized H_2_O (11.5 mL) in a 25 mL round‐bottomed flask. NaBH4 (6.1 mmol) was dispersed in deionized H_2_O (1.5 mL) for 1 min and then added to the cellobiose solution. The reaction mixture was stirred (600 rpm) at room temperature for 1 h. Cellobiitol was isolated by using the same work‐up procedure as for xylobiitol. The final product was dried in a vacuum oven under 50 mbar at 40 °C for 24 h, resulting in a white solid (71 % yield). The product was characterized by 2D HSQC NMR in [D_6_]DMSO, indicating no impurities (Figure S12). The ^13^C−^1^H correlation signals assignment of the product is given in Table S4.

## Conflict of interest

The authors declare no conflict of interest.

1

## Supporting information

As a service to our authors and readers, this journal provides supporting information supplied by the authors. Such materials are peer reviewed and may be re‐organized for online delivery, but are not copy‐edited or typeset. Technical support issues arising from supporting information (other than missing files) should be addressed to the authors.

Supporting Information

## Data Availability

Research data are not shared.
