# Supplementary material for: Lignin Stabilization and Carbohydrate Nature in H‐transfer Reductive Catalytic Fractionation: The Role of Solvent Fractionation of Lignin Oil in Structural Profiling
Source: ChemSusChem. 2022 Dec 21;16(3):e202201875. doi: 10.1002/cssc.202201875 (PMC10108069; doi:10.1002/cssc.202201875)
Supplement: Supplementary file 1 — Supporting Information [file CSSC-16-0-s001.pdf]

ChemSusChem

Supporting Information

Lignin Stabilization and Carbohydrate Nature in H-transfer Reductive Catalytic Fractionation: The Role of Solvent Fractionation of Lignin Oil in Structural Profiling**

Raul Rinken, Dean Posthuma, and Roberto Rinaldi*© 2022 The Authors. ChemSusChem published by Wiley-VCH GmbH. This is an open access article under the terms of the Creative Commons Attribution License, which permits use, distribution and reproduction in any medium, provided the original work is properly cited.

Supporting Information

Contents

Semiquantitative estimation of structural features in lignin and carbohydrate

Table S1. Hildebrand solubility parameter (δ) and HSP (δ_D, δ_P, δ_H) for lignin-derived monophenolics.

Table S2. Hildebrand solubility parameter (δ) and HSP (δ_D, δ_P, δ_H) for lignins.

Figure S1. Ternary diagram showing the distribution of theoretical values of H/C and O/C in the whole range of S/G/H ratios. Values calculated for hypothetical linear lignin structures connected by β-O-4. The dot indicates the hypothetical H/C and O/C molar ratio values considered as the theoretical values for the Poplar lignin in this study.

Figure S2. HSQC NMR of RCF lignin oil zoomed in (A) aliphatic region, (B) oxygenated aliphatic region, (C) anomeric region, and (D) aromatic region (solvent: DMSO-d_6).

Figure S3. HSQC NMR spectrum of RCF lignin oil methanol-insoluble fraction zoomed in (A) aliphatic region, (B) oxygenated aliphatic region, (C) anomeric region, and (D) aromatic region (solvent: DMSO-d_6). The ^{1}H-^{13}C correlation signals for the $A_{(CH_2)}$ linkages were below the threshold chosen to present the HSQC NMR spectral data. Captions for signals not detected were greyed out.

Figure S4. HSQC NMR spectrum of RCF lignin oil methanol-soluble fraction zoomed in (A) aliphatic region, (B) oxygenated aliphatic region, (C) anomeric region, and (D) aromatic region (solvent: DMSO-d_6).

Figure S5. HSQC NMR spectrum of RCF lignin oil acetone-insoluble fraction zoomed in (A) aliphatic region, (B) oxygenated aliphatic region, (C) anomeric region, and (D) aromatic region (solvent: DMSO-d_6). The ^{1}H-^{13}C correlation signals for the $A_{(CH_2)}$ linkages were below the threshold chosen to present the HSQC NMR spectral data. Captions for signals not detected were greyed out.

Figure S6. HSQC NMR spectrum of RCF lignin oil acetone-soluble fraction zoomed in (A) aliphatic region, (B) oxygenated aliphatic region, (C) anomeric region, and (D) aromatic region (solvent: DMSO-d_6). Captions for signals not detected were greyed out.

Figure S7. HSQC NMR spectrum of RCF lignin oil EtOAc-insoluble fraction zoomed in (A) aliphatic region, (B) oxygenated aliphatic region, (C) anomeric region, and (D) aromatic region (solvent: DMSO-d_6). Captions for signals not detected were greyed out.

Figure S8. HSQC NMR spectrum of RCF lignin oil EtOAc-soluble fraction zoomed in (A) aliphatic region, (B) oxygenated aliphatic region, (C) anomeric region, and (D) aromatic region (solvent: DMSO-d_6). Captions for signals not detected were greyed out.

Figure S9. Results from hot water extraction carried out on the acetone-insoluble fraction of the RCF lignin oil. HSQC NMR spectrum of hot water extractives presented in (A) aliphatic region, (B) oxygenated aliphatic region, (C) anomeric region, (D) oxygenated aliphatic region focused on carbohydrates, and (E) aromatic region (solvent: DMSO-d_6). Captions for signals not detected were greyed out.

Figure S10. Results from hot water extraction carried out on the acetone-insoluble fraction of the RCF lignin oil. HSQC NMR spectrum of hot water-insoluble fraction presented in (A) aliphatic region, (B) oxygenated aliphatic region, (C) anomeric region, and (E) aromatic region (solvent: DMSO-d_6). The ^{1}H-^{13}C correlation signals for the $A_{(CH_2)}$ linkages were below the threshold chosen to present the HSQC NMR spectral data. Captions for signals not detected were greyed out.

Scheme S1. Notation for the interunit bonding motifs for lignin and oligosaccharide structural models.

Table S3. List of ^{13}C-^{1}H pairs with their respective δC/δH values (in ppm) obtained from the spectra in this study (solvent: DMSO-d_6).

Figure S11. HSQC NMR spectra (A) xylobiose and (B) cellobiose (solvent: DMSO-d_6).

Table S4. List of ^{13}C-^{1}H pairs with their respective δC/δH values (in ppm) obtained from the spectra of xylobiose and cellobiose in this study (solvent: DMSO-d_6).

Figure S12. HSQC NMR spectra (A) xylobiitol and (B) cellobiitol (solvent: DMSO-d_6).

Table S5. List of ^{13}C-^{1}H pairs with their respective δC/δH values (in ppm) obtained from the spectra of xylobiitol and cellobiitol in this study (solvent: DMSO-d_6).

Figure S13. Correlation between the %PB units relative to combined G- and S-unit content and the apparent $\overline{M}_w$ value of the fractions isolated by solvent fractionation of the RCF lignin oil.

References

Semiquantitative estimation of structural features in lignin and carbohydrate

Lignin linkage count per 100 aromatic units, the relative abundance of reduced β-O-4 linkages and different aromatic unit content were estimated by Equations S1-S7:

$$\text{Linkage count per 100 aromatic units (\%)} = \frac{V(C_\alpha)_{\text{linkage}}}{0.5V(S_{2/6})+V(G_2)} \times 100 \qquad \text{S1}$$

$$\text{Reduced β-O-4 linkages (\%)} = \frac{V(C_{\beta(CH_2)(SS)})+V(C_{\beta(CH_2)(SG)})}{V(C_{\beta(S)})+V(C_{\beta(G)})+V(C_{\beta(CH_2)(SS)})+V(C_{\beta(CH_2)(SG)})} \times 100 \qquad \text{S2}$$

$$\text{Reduced β-O-4 linkage count per 100 aromatic units} = \frac{V(C_\alpha)_{\beta\text{-O-4}}}{0.5V(S_{2/6})+V(G_2)} \times \frac{[\text{Reduced β-O-4 linkages (\%)}]}{100-[\text{Reduced β-O-4 linkages (\%)}]} \times 100 \qquad \text{S3}$$

$$\text{S-unit (\%)} = \frac{0.5V(S_{2/6})}{0.5V(S_{2/6})+V(G_2)} \times 100 \qquad \text{S4}$$

$$\text{G-unit (\%)} = \frac{V(G_2)}{0.5V(S_{2/6})+V(G_2)} \times 100 \qquad \text{S5}$$

$$\text{H-unit (\%)} = \frac{0.5V(H_{2/6})}{0.5V(S_{2/6})+V(G_2)+0.5V(H_{2/6})} \times 100 \qquad \text{S6}$$

$$\text{PB-unit (\%)} = \frac{0.5V(PB_{2/6})}{0.5V(S_{2/6})+V(G_2)} \times 100 \qquad \text{S7}$$

The average degree of polymerization (DP) of the main oligoxylan backbone in oligo(arabino)xylan alcohols, and the count of arabinose units decorating the oligoxylan backbone were estimated by Equations S8-S9:

$$\text{DP(oligoxylan alcohol backbone)} = \frac{V(X1'_I)+V(X1_{NR/I})}{V(X1'_I)} \times 100 \qquad \text{S8}$$

$$\text{Arabinose unit in oligo(arabino)xylan alcohol} = \frac{V(\text{Ara1})}{V(X1'_I)+V(X1_{NR/I})} \times 100 \qquad \text{S7}$$

where V denotes the volume integral for the indicated ^{13}C-^{1}H pair in the HSQC NMR spectra. The volume integral for the indicated ^{13}C-^{1}H pairs was measured five times on different occasions in order to determine the standard deviation.

Notes:

1. The $^{1}J_{CH}$ dependence of polarisation transfer in HSQC experiments is not entirely suppressed in regular HSQC pulse sequences (e.g., Bruker *hsqcetgpsi*).).[1] As a result, the response for ^{13}C-^{1}H pairs is not entirely uniform in the entire spectral range. Despite this, regular HSQC NMR measurements offer can serve for the estimation of semiquantitative information for the characterization and comparison of lignins when ^{1}H–^{13}C pairs in a similar chemical environment are chosen (*e.g..,* C_{α}–H_{α} signals for the side-chain of lignin units or the C2–H2 and C6–H6 pairs in the G- and S-lignin units). In this case, the $^{1}J_{CH}$ values for the specific entities are very similar. Nonetheless, a systematic deviation owing to the non-universal response for ^{13}C-^{1}H pairs is present in the estimates.
2. In Equation 2, we decided to use the volume integral of C_{β} in the determination of the percentual of reduced β-O-4 linkages in order to mitigate any effect of $^{1}J_{CH}$ on the signal response uniformity.. Since native and reduced β-O-4 linkages present identical neighboring atoms connected on the C_{β} atom, $^{1}J_{CH}$ values should be identical. As a result, the signal response is reasonably similar for the ^{13}C-^{1}H pairs in the HSQC NMR spectrum.
3. In Equations 1, 4, 6, and 7, the contribution of $H_{2/6}$ was excluded from the denominator since the H-units signal seems to come from products of guaiacol demethoxylation. Notably, the %H-unit in the low-MW soluble fractions was <0.2%.
4. In Equations S1, S4, S6, and S7, the contribution of $H_{2/6}$ was excluded from the denominator since the H-units signal seems to come from products of guaiacol demethoxylation. Notably, the %H-unit in the low-MW soluble fractions was <0.2%.

Table S1. Hildebrand solubility parameter (δ) and HSP (δ_D, δ_P, δ_H) for lignins.

Lignin source	Extraction conditions	δ ($MPa^{1/2}$)	δ_D ($MPa^{1/2}$)	δ_P ($MPa^{1/2}$)	δ_H ($MPa^{1/2}$)	R_o ($MPa^{1/2}$)
Spruce [2,3]	Björkman milled wood	31.0	21.9	14.1	16.9	13.7
Eucalyptus [4]	Kraft pulping	28.3	17.8	18.4	12.1	13.5
Pine [4]	Kraft pulping	24.6	16.7	13.7	11.7	NR<br>-

Table S2. Hildebrand solubility parameter (δ) and HSP (δ_D, δ_P, δ_H) for lignin-derived monophenolics available in the literature.

**Compound**	**δ (MPa$^{1/2}$)**	**δ_D (MPa$^{1/2}$)**	**δ_P (MPa$^{1/2}$)**	**δ_H (MPa$^{1/2}$)**
Phenol [5]	24.1	18.0	5.9	14.9
4-Methylphenol [6]	22.5	17.0	4.3	14.1
4-Ethylphenol [5]	23.7	19.2	5.3	12.8
p-Coumaryl alcohol [5]	26.7	19.1	7.0	17.3
Guaiacol [5]	23.8	18.0	8.2	13.3
4-Methylguaiacol[6]	22.1	17.0	6.6	12.5
Eugenol[5]	24.2	19.0	7.5	13.0
p-Coniferyl alcohol[5]	26.0	19.0	7.3	16.1
Syringol[5]	24.9	19.3	7.6	13.7
p-Sinapyl alcohol[5]	26.1	19.2	7.3	16.1

Calculation of theoretical H/C and O/C values for hypothetical hardwood lignin

Hypothetical hardwood lignin made of β-O-4 linkages was considered to determine the effect of S/G/H ratio on the theoretical values for H/C and O/C ratios. An aromatic unit linked on both ends has 11 carbon, 5 oxygen, and 14 hydrogen atoms for S-unit; 10 carbon, 4 oxygen, and 12 hydrogen atoms for G-unit; 9 carbon, 3 oxygen, and 10 hydrogen atoms for H unit. We assumed a degree of polymerization above 10, allowing us to neglect the impact of the end groups on the theoretical values for H/C and O/C molar ratios. They were estimated by Equations 8 and 9.

$$H/C = \frac{14\times(S_{unit}) + 12\times(G_{unit}) + 10\times(H_{unit})}{11\times(S_{unit}) + 10\times(G_{unit}) + 9\times(H_{unit})} \quad \mathbf{8}$$

$$O/C = \frac{5\times(S_{unit}) + 4\times(G_{unit}) + 3\times(H_{unit})}{11\times(S_{unit}) + 10\times(G_{unit}) + 9\times(H_{unit})} \quad \mathbf{9}$$

where S_{unit}, G_{unit} and H_{unit} represent the fraction of each unit present in hardwood. For poplar containing 62% of S-type lignin (S_{unit}=0.62) and 38% of G-type (G_{unit}=0.38), the theoretical values for H/C ratio and O/C ratio are 1.25 and 0.44, respectively. Figure S1 displays corresponding H/C and O/C molar ratios for different S/G/H ratios under the same assumptions, with the highlighted spot for poplar used in this study. For hypothetical SG lignin structures with G-unit fraction between 0.25 and 0.50, the values for H/C and O/C were 1.25±0.02 and 0.44±0.02, respectively.

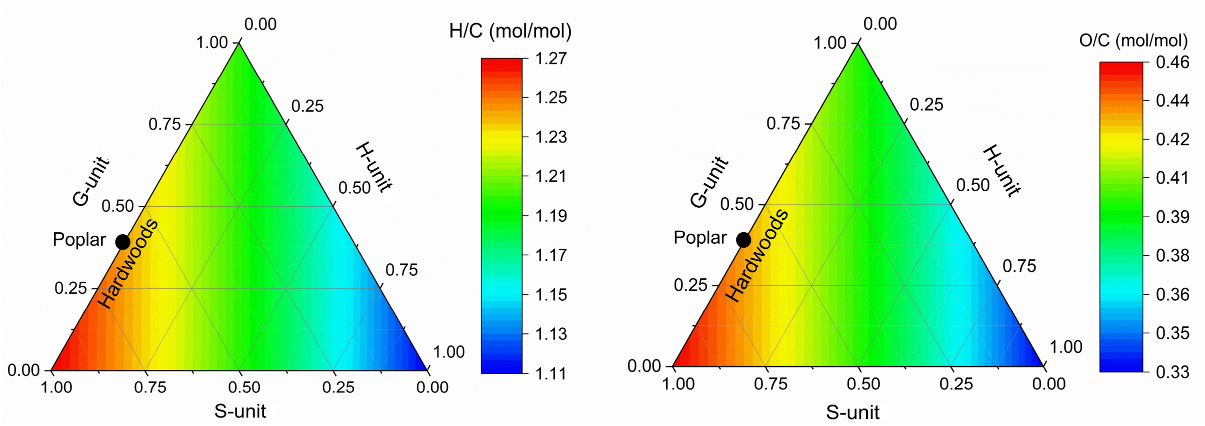

Figure S1. Ternary diagram showing the distribution of theoretical values of H/C and O/C in the whole range of S/G/H ratios. Values calculated for hypothetical linear lignin structures connected by β-O-4. The dot indicates the hypothetical H/C and O/C molar ratio values considered as the theoretical values for the Poplar lignin in this study.

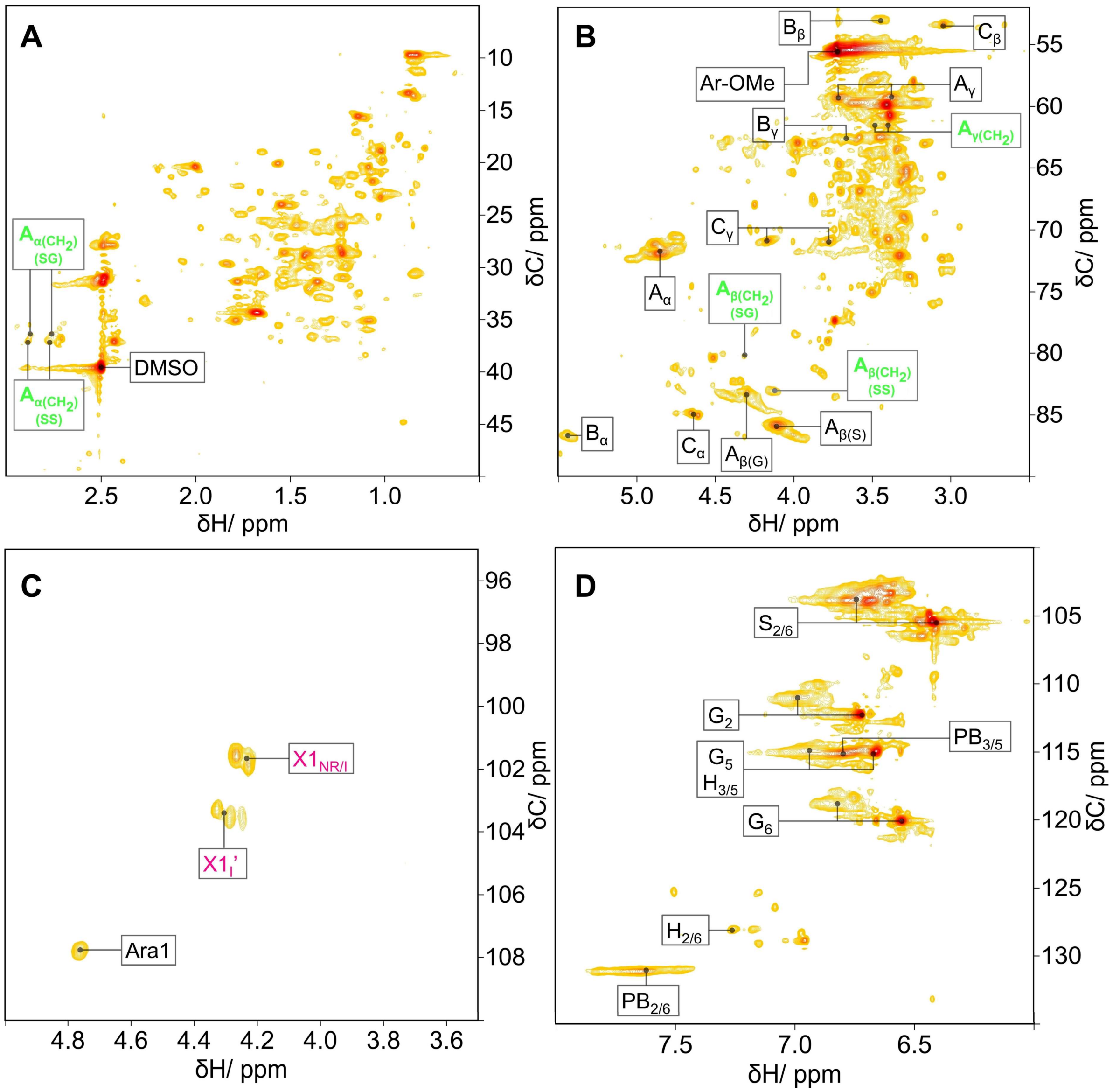

Figure S2. HSQC NMR spectrum of RCF lignin oil zoomed in (A) aliphatic region, (B) oxygenated aliphatic region, (C) anomeric region and (D) aromatic region (solvent: DMSO-d_6).

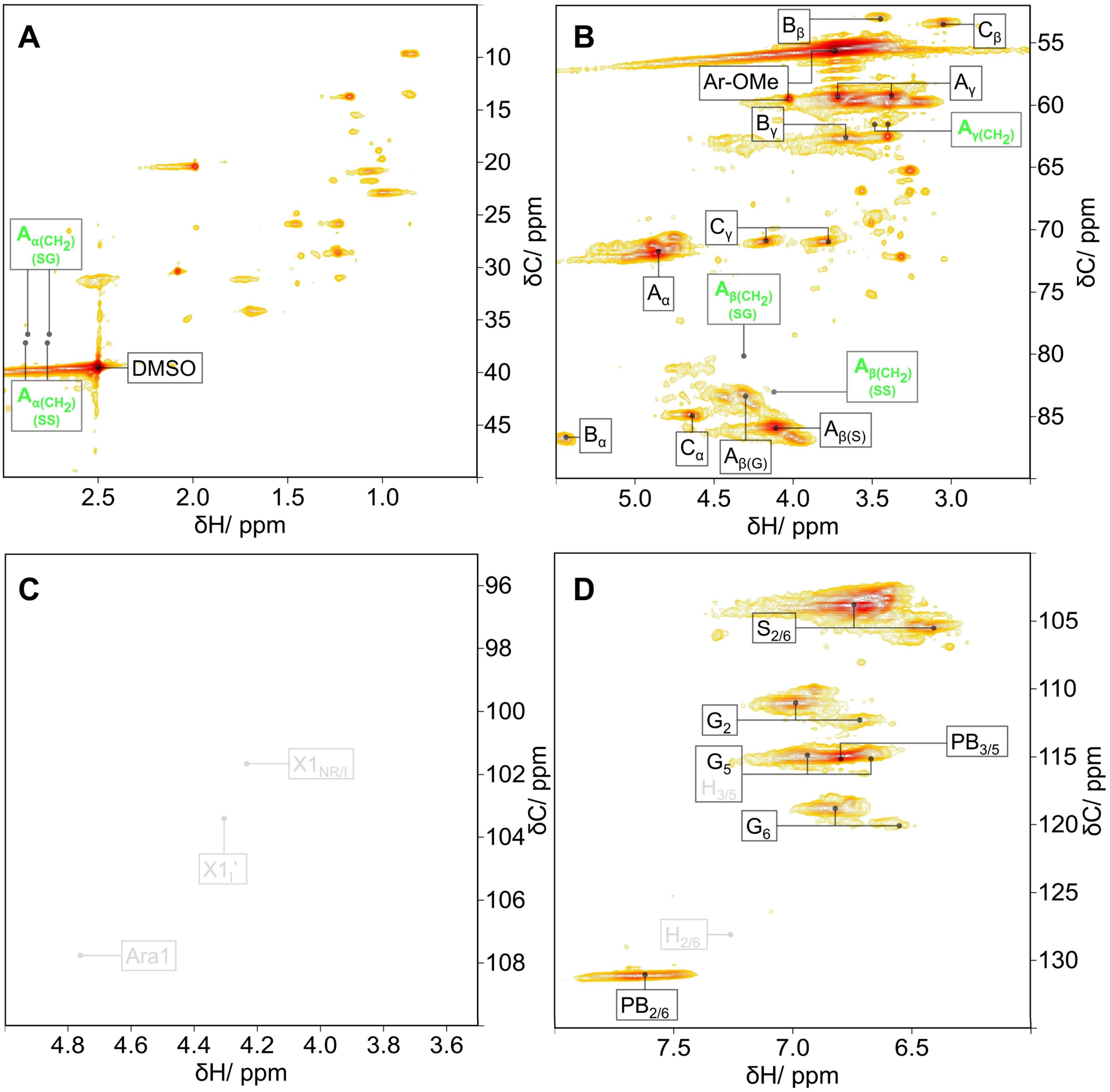

Figure S3. HSQC NMR spectrum of RCF lignin oil methanol-insoluble fraction zoomed in (A) aliphatic region, (B) oxygenated aliphatic region, (C) anomeric region and (D) aromatic region (solvent: DMSO-d_6). Note: The ^{1}H-^{13}C correlation signals for the $A_{(CH_2)}$ linkages were below the threshold chosen to present the HSQC NMR spectral data. Captions for signals not detected were greyed out.

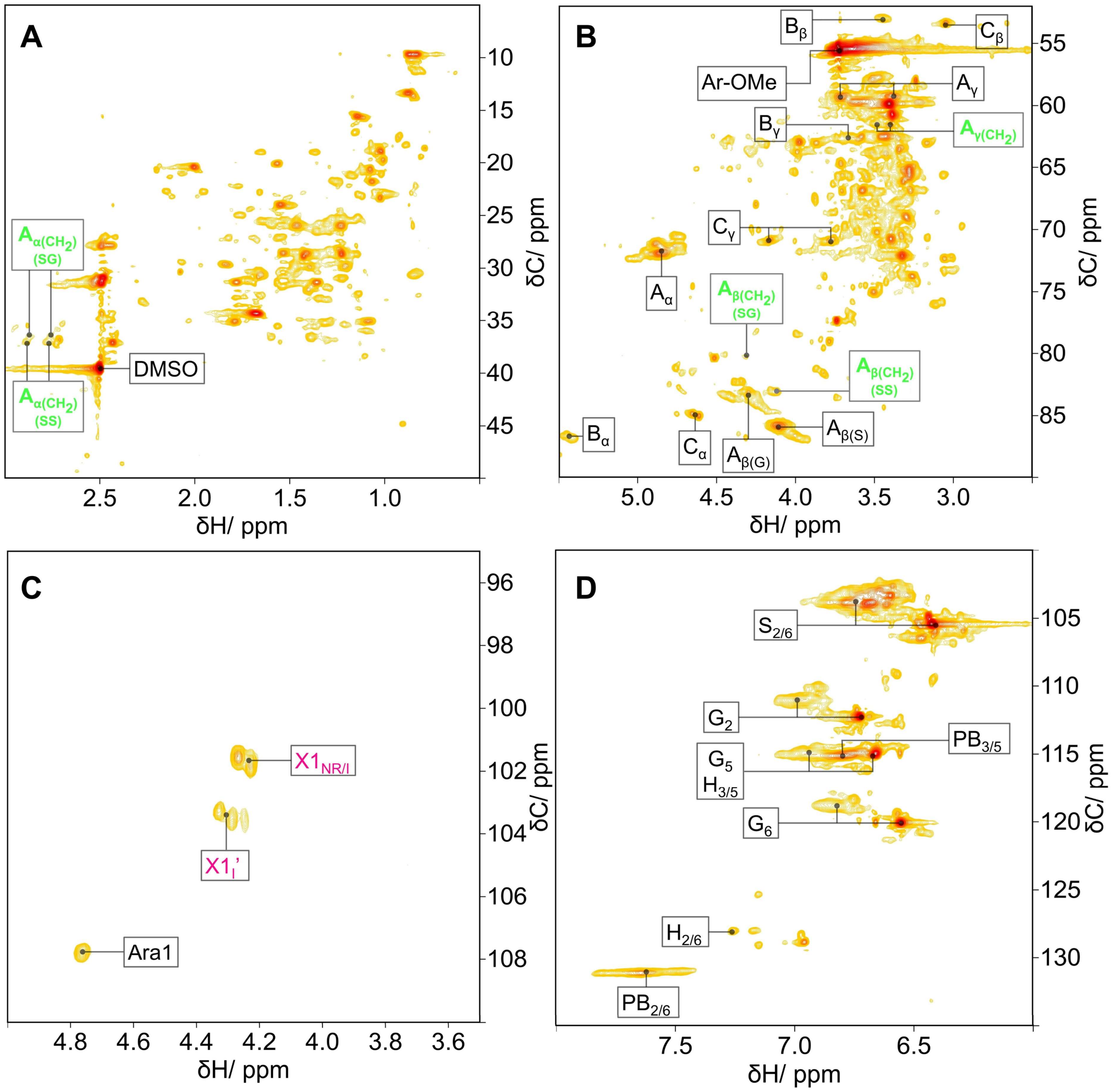

Figure S4. HSQC NMR spectrum of RCF lignin oil methanol-soluble fraction zoomed in (A) aliphatic region, (B) oxygenated aliphatic region, (C) anomeric region and (D) aromatic region (solvent: DMSO-d_6).

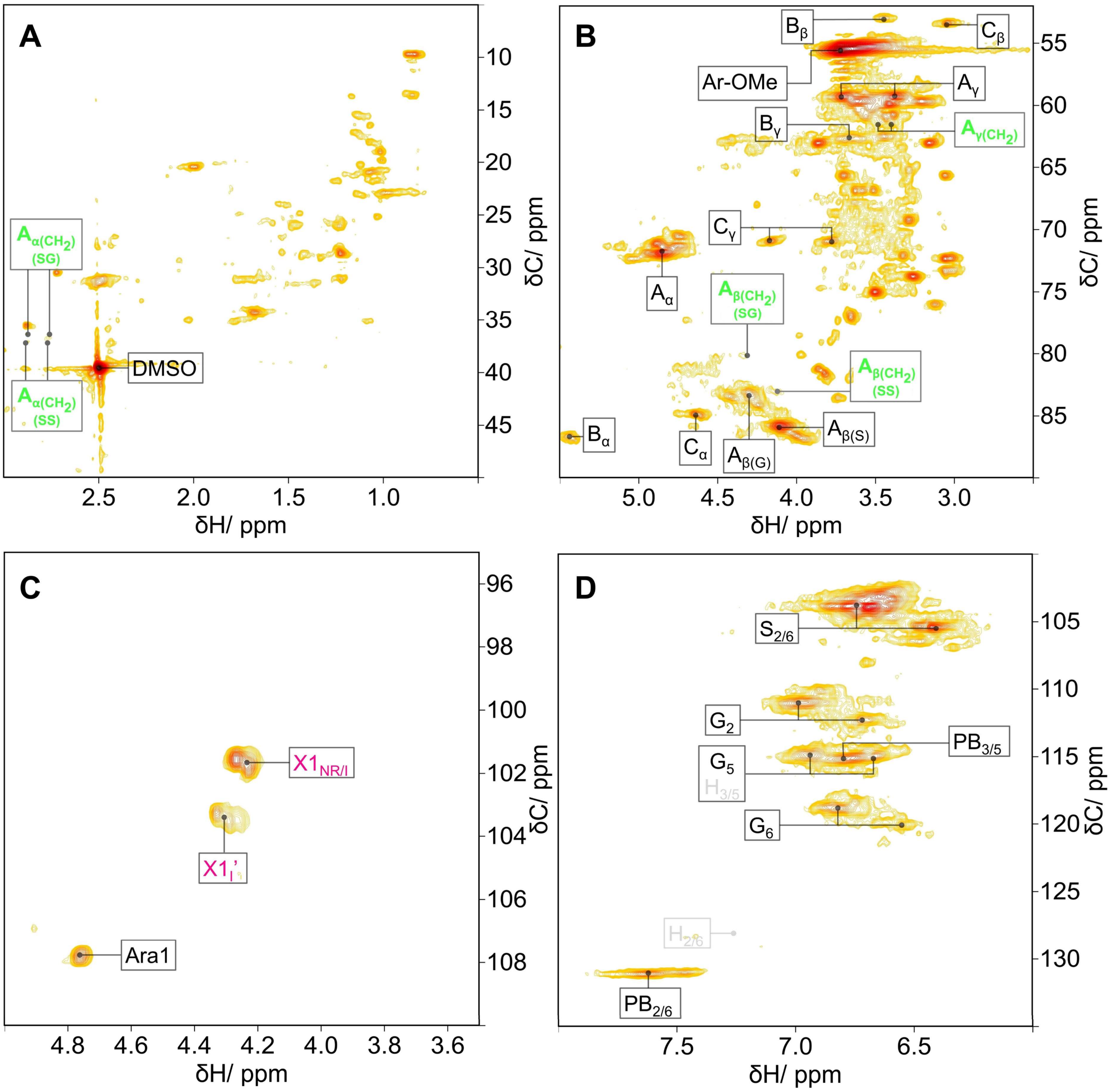

Figure S5. HSQC NMR spectrum of RCF lignin oil acetone-insoluble fraction zoomed in (A) aliphatic region, (B) oxygenated aliphatic region, (C) anomeric region and (D) aromatic region (solvent: DMSO-*d*6). The ^{1}H-^{13}C correlation signals for the $A_{(CH_2)}$ linkages were below the threshold chosen to present the HSQC NMR spectral data. Captions for signals not detected were greyed out.

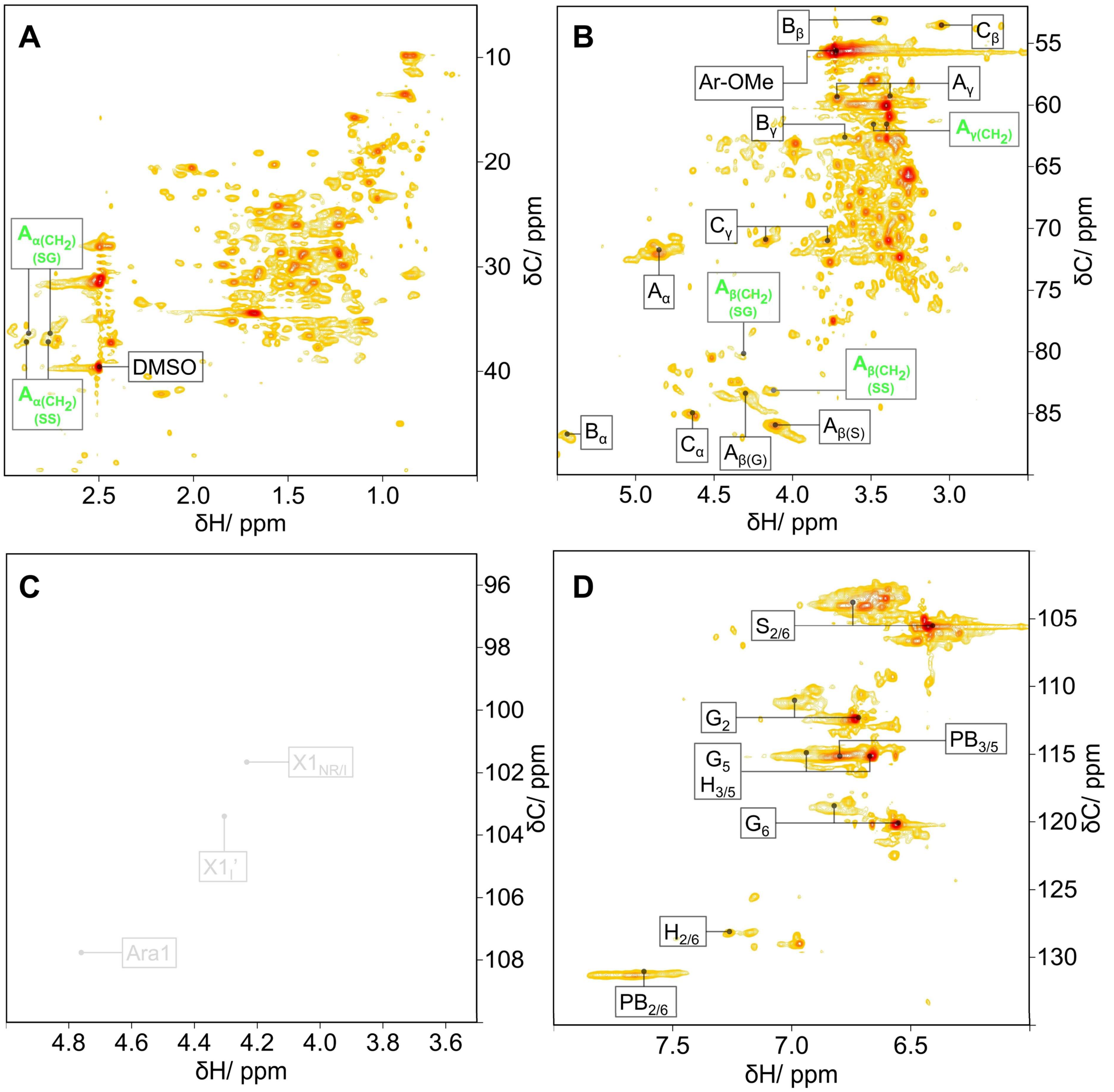

Figure S6. HSQC NMR spectrum of RCF lignin oil acetone-soluble fraction zoomed in (A) aliphatic region, (B) oxygenated aliphatic region, (C) anomeric region and (D) aromatic region (solvent: DMSO-d_6). Captions for signals not detected were greyed out.

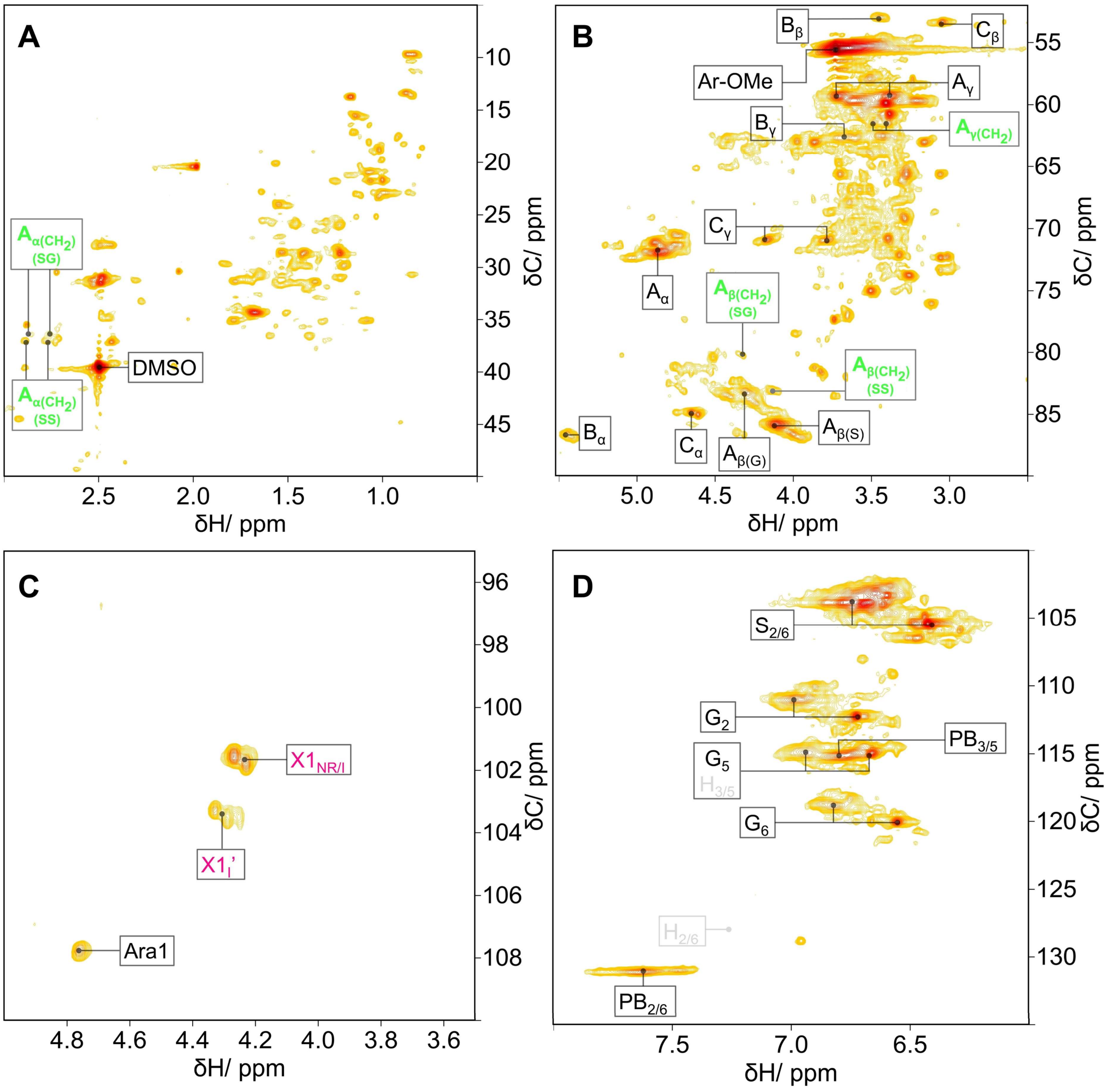

Figure S7. HSQC NMR spectrum of RCF lignin oil EtOAc-insoluble fraction zoomed in (A) aliphatic region, (B) oxygenated aliphatic region, (C) anomeric region and (D) aromatic region (solvent: DMSO-*d*$_6$). Captions for signals not detected were greyed out.

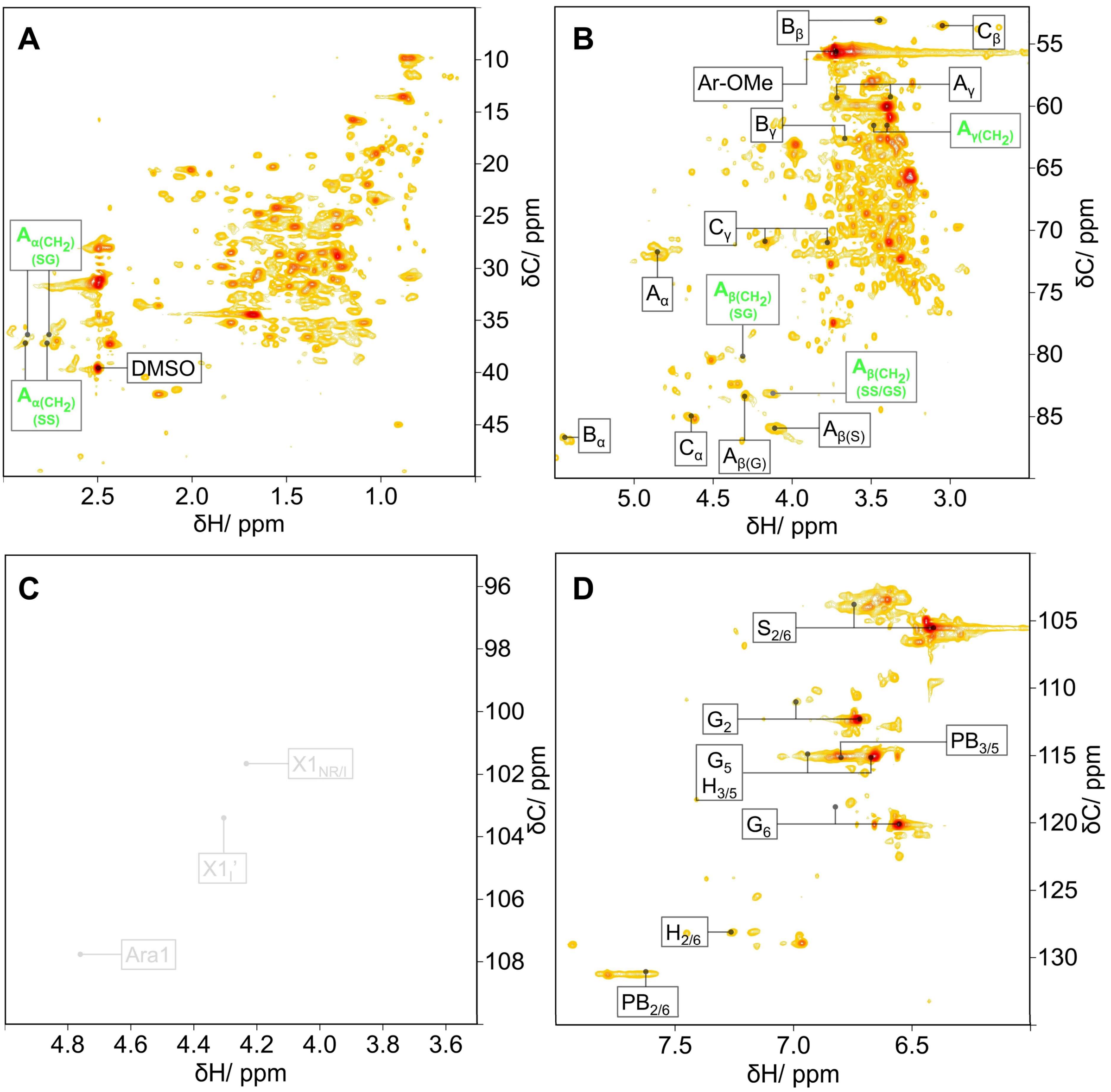

Figure S8. HSQC NMR spectrum of EtOAc-soluble fraction of RCF lignin oil zoomed in (A) aliphatic region, (B) oxygenated aliphatic region, (C) anomeric region, and (D) aromatic region. (solvent: DMSO-d_6). Captions for signals not detected were greyed out.

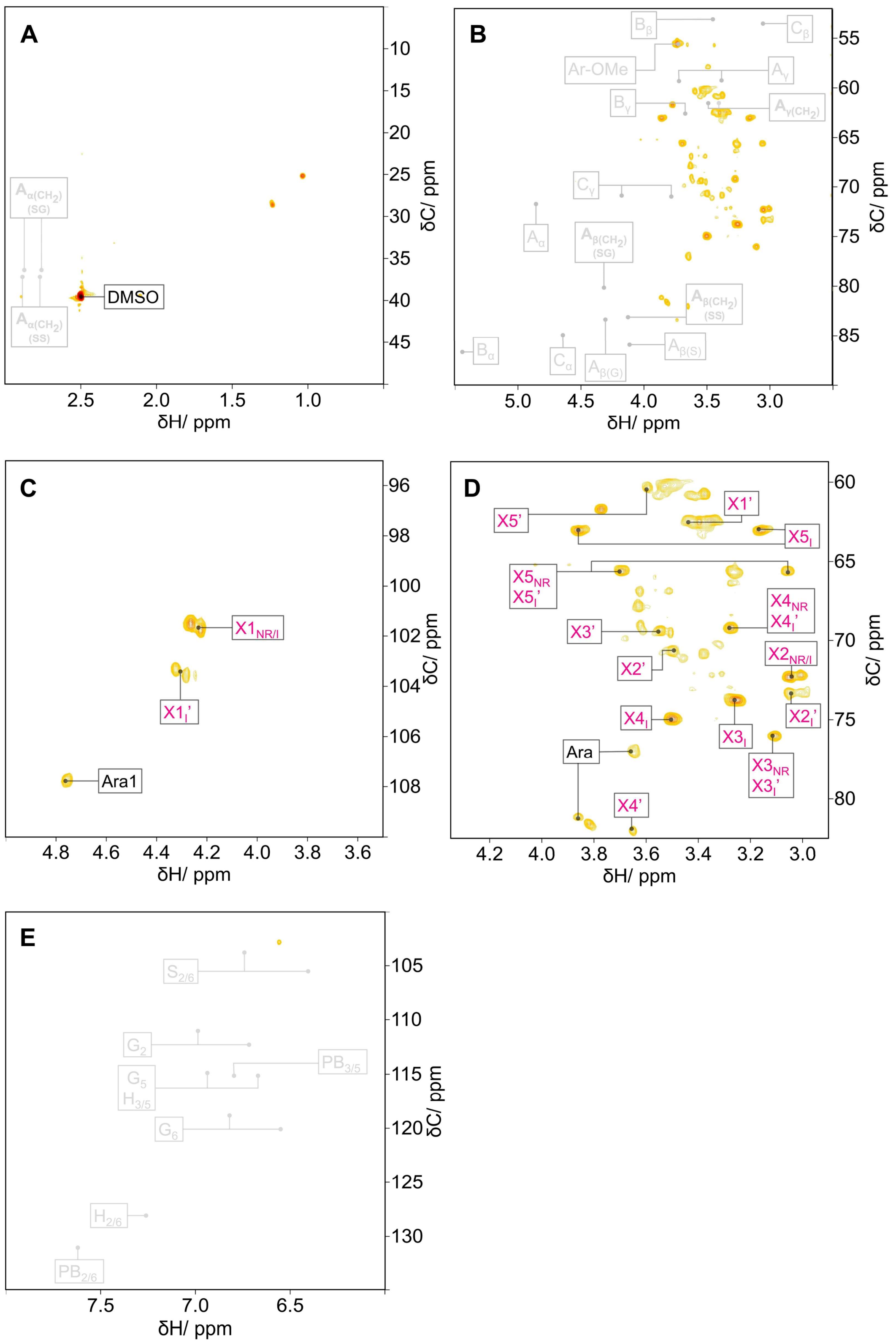

Figure S9. Results from the hot water extraction carried out on the acetone-insoluble fraction of the RCF lignin oil. HSQC NMR spectrum of hot water extractives presented in (A) aliphatic region, (B) oxygenated aliphatic region, (C) anomeric region, (D) oxygenated aliphatic region focused on carbohydrates, and (E) aromatic region (solvent: DMSO-d_6). Captions for signals not detected were greyed out.

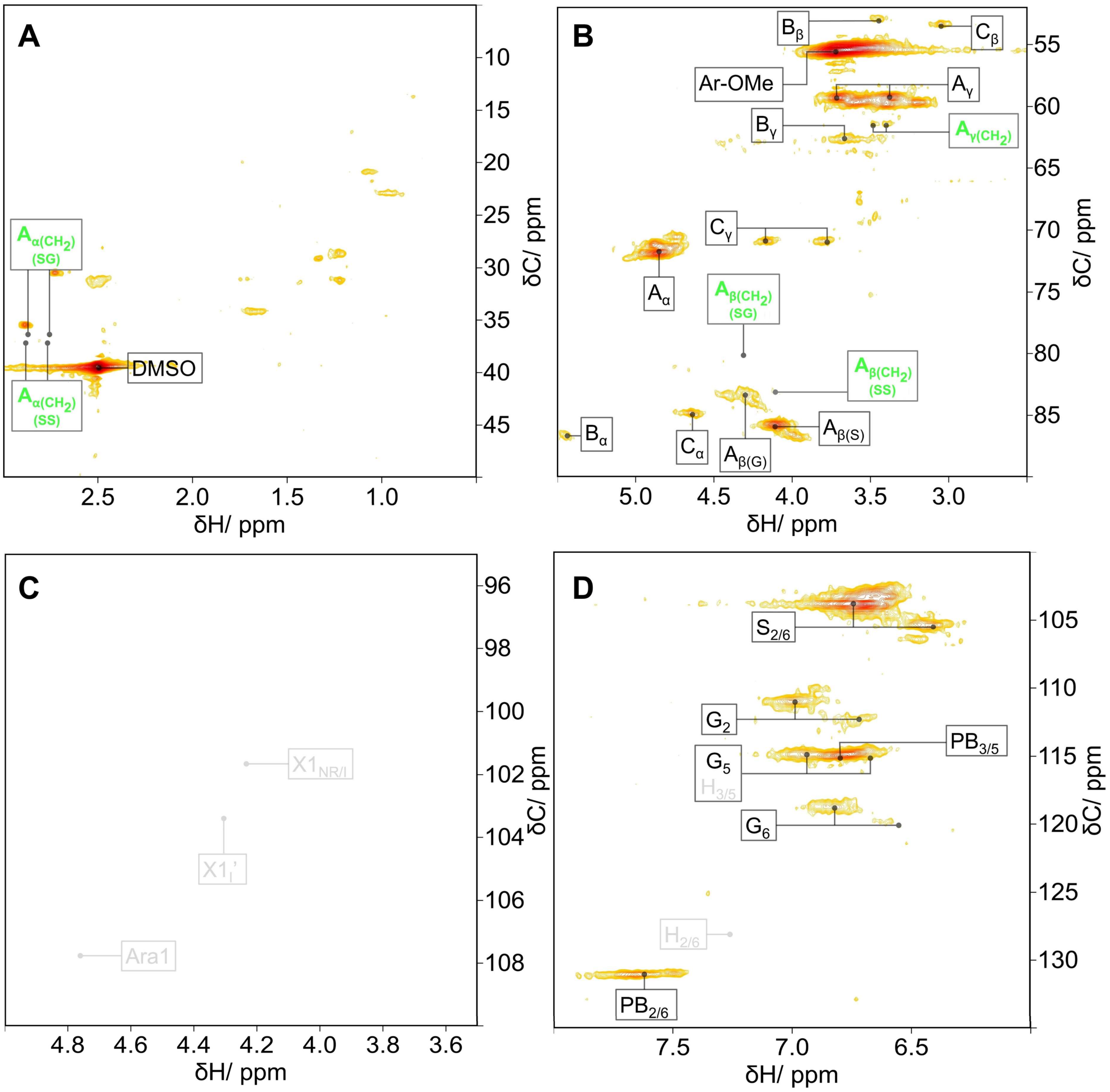

Figure S10. Results from the hot water extraction carried out on the acetone-insoluble fraction of the RCF lignin oil. HSQC NMR spectrum of hot water-insoluble fraction presented in (A) aliphatic region, (B) oxygenated aliphatic region, (C) anomeric region, and (E) aromatic region (solvent: DMSO-*d*$_6$). The ^{1}H-^{13}C correlation signals for the $A_{(CH_2)}$ linkages were below the threshold chosen to present the HSQC NMR spectral data. Captions for signals not detected were greyed out.

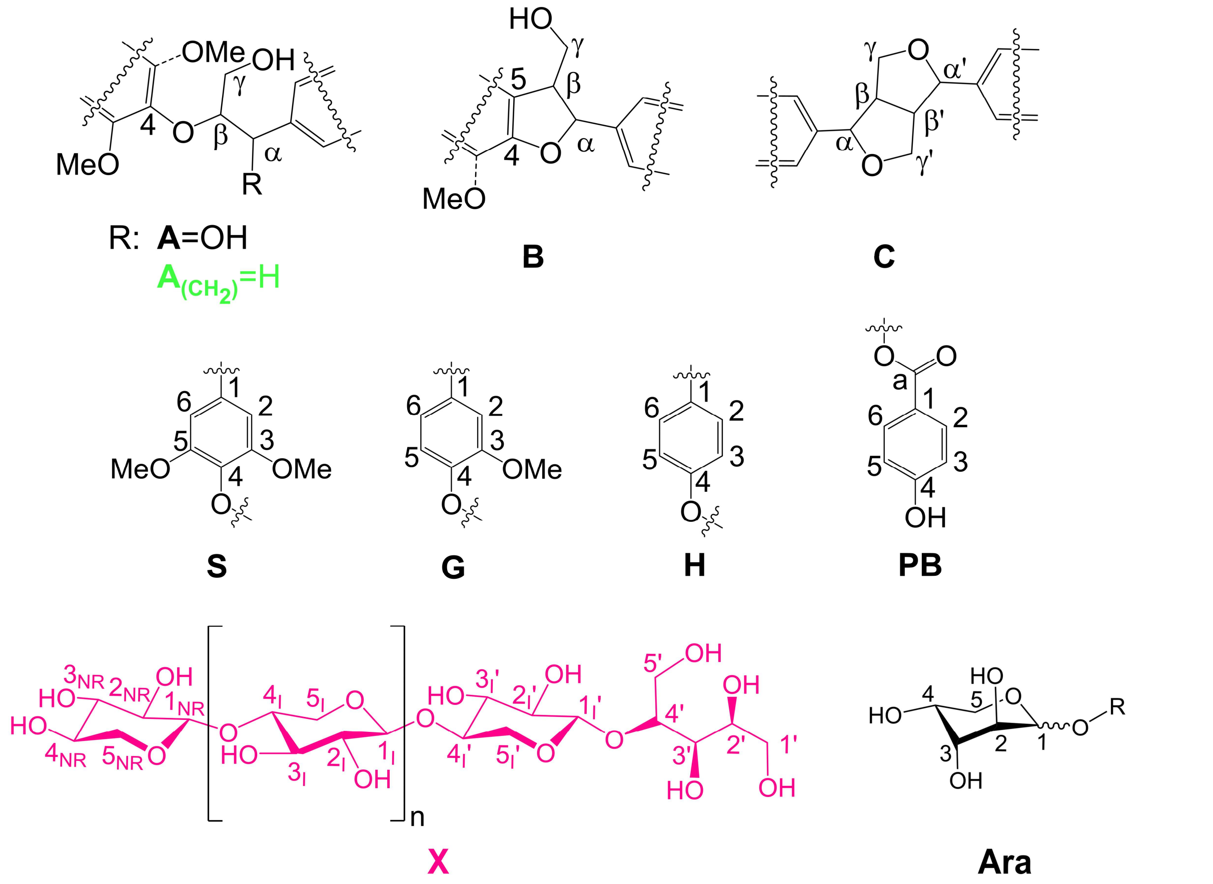

Scheme S1. Notation for the interunit bonding motifs for lignin and oligosaccharide structural models.

Table S3. List of ^{13}C-^{1}H pairs with their respective δC/δH values (in ppm) obtained from the spectra in this study (solvent: DMSO-d_6).

**Label**	**δC/δH (ppm)**	**Description**
$\mathbf{A_{\alpha}}$	71.90/4.86	C_{α}-H_{α} in β-O-4 lignin linkages
$\mathbf{A_{(CH_2)\alpha\ (SG)}}$	36.53/2.86<br>36.53/2.78	C_{α}-H_{α} in reduced β-O-4 lignin linkages containing S and G units (with etherified G unit)
$\mathbf{A_{(CH_2)\alpha(SS)}}$	37.06/2.90<br>37.06/2.78	C_{α}-H_{α} reduced β-O-4 bonding motif formed between two S units
$\mathbf{A_{(G)\beta}}$	83.34/4.27	C_{β}-H_{β} in β-O-4 bonding motif (with etherified G unit)
$\mathbf{A_{(S)\beta}}$	85.81/4.10	C_{β}-H_{β} in β-O-4 bonding motif (with etherified S unit)
$\mathbf{A_{(CH_2)\beta\ (SG)}}$	80.56/4.30	C_{β}-H_{β} in reduced β-O-4 bonding motif formed between S and G units (with etherified G unit)
$\mathbf{A_{(CH_2)\beta\ (SS)}}$	83.32/4.10	C_{β}-H_{β} in reduced β-O-4 bonding motif formed between S units
$\mathbf{A_{(CH_2)\gamma}}$	61.34/3.39<br>61.34/3.48	C_{γ}-H_{γ} in reduced β-O-4 bonding motif
$\mathbf{A_{\gamma}}$	59.23/3.38<br>59.23/3.71	C_{γ}-H_{γ} in β-O-4 bonding motif
$\mathbf{B_{\alpha}}$	86.69/5.45	C_{α}-H_{α} in phenylcoumaran bonding motif
$\mathbf{B_{\beta}}$	53.07/3.45	C_{β}-H_{β} in phenylcoumaran bonding motif
$\mathbf{B_{\gamma}}$	62.75/3.68	C_{γ}-H_{γ} in phenylcoumaran bonding motif
$\mathbf{C_{\alpha}}$	85.28/4.62	C_{α}-H_{α} in resinol bonding motif
$\mathbf{C_{\beta}}$	53.60/3.05	C_{β}-H_{β} in resinol bonding motif
$\mathbf{C_{\gamma}}$	70.85/3.87<br>70.85/4.16	C_{γ}-H_{γ} in resinol bonding motif

HSQC NMR spectra of xylobiose and cellobiose

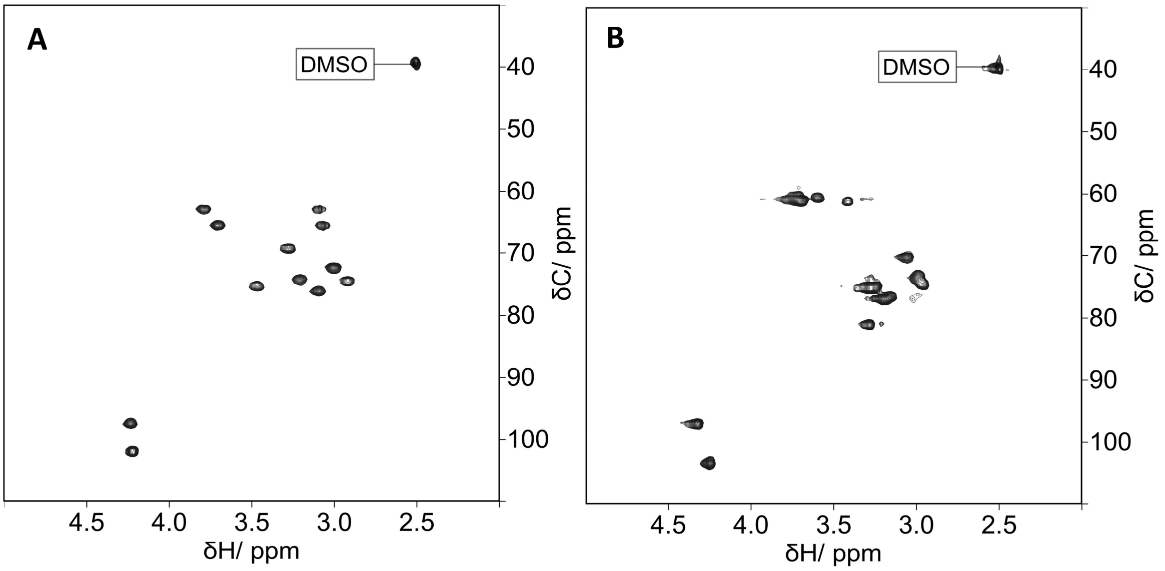

Figure S11. HSQC NMR spectra (A) xylobiose and (B) cellobiose. (solvent: DMSO-d_6).

Table S4. List of ^{13}C-^{1}H pairs with their respective δC/δH values (in ppm) obtained from the spectra of xylobiose and cellobiose in this study (solvent: DMSO-d_6).

Carbon atom	Xylobiose	Cellobiose
	δC/δH (ppm)	δC/δH (ppm)
1	101.56/4.22	103.56/4.25
2	72.21/3.00	73.54/2.99
3	76.21/3.09	76.88/3.16
4	69.54/3.28	70.21/3.06
5	65.54/(3.06/3.71)	76.88/3.16
6	N/A	61.30/(3.41/3.70)
1′	97.56/4.23	96.89/4.32
2′	74.21/2.92	74.88/2.95
3′	74.21/3.21	75.54/3.27
4′	75.54/3.47	80.88/3.28
5′	62.87<br>3.09/3.80	75.54<br>3.27
6′	N/A	60.87/(3.60/3.71)

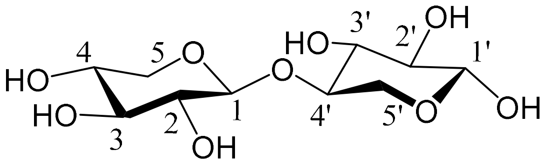

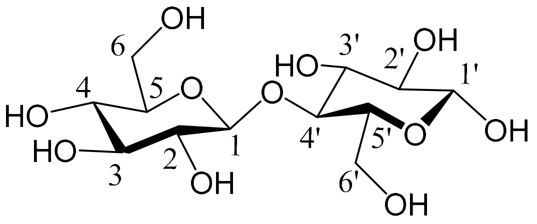

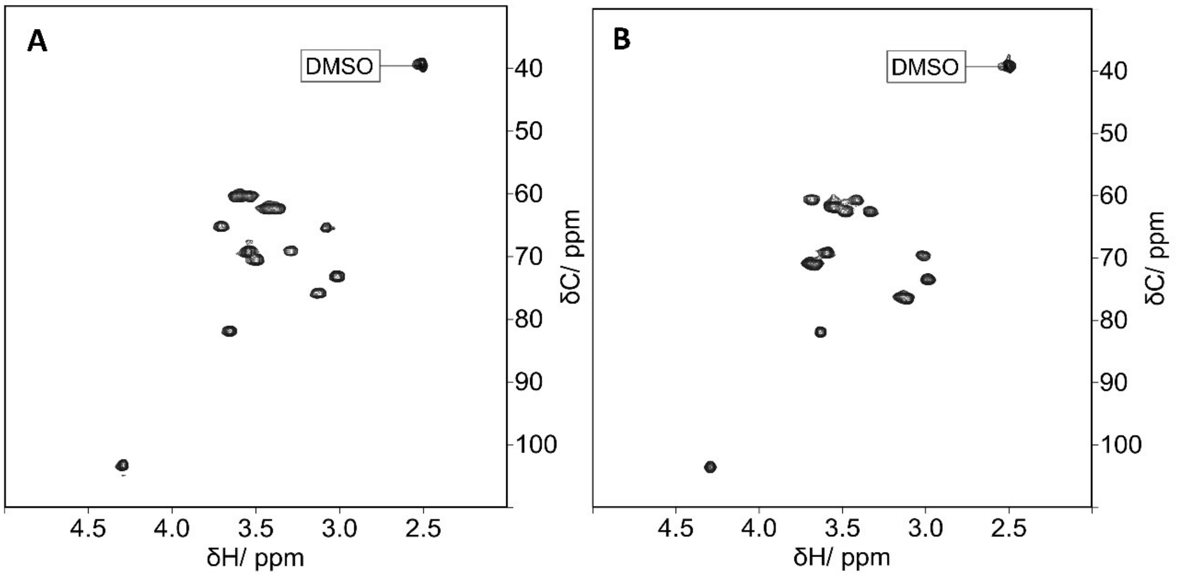

Figure S12. HSQC NMR spectra (A) xylobiitol and (B) cellobiitol (solvent: DMSO-d_6).

Table S5. List of ^{13}C-^{1}H pairs with their respective δC/δH values (in ppm) obtained from the spectra of xylobiitol and cellobiitol in this study (solvent: DMSO-d_6).

Carbon atom	Xylobiitol	Cellobiitol
	δC/δH (ppm)	δC/δH (ppm)
1	103.56/4.29	103.56/4.30
2	72.88/3.02	73.54/2.98
3	76.21/3.13	75.80/3.14
4	68.87/3.29	69.54/3.02
5	65.54/ 3.09/3.71	76.21/3.13
6	-	60.87/ 3.41/3.68
1′	62.20/3.43	62.87/ 3.33/3.48
2′	70.87/3.50	70.87/3.68
3′	69.54/3.54	68.87/3.59
4′	82.22/3.65	81.55/3.63
5′	60.20/3.62	70.87/3.68
6′	-	61.54/3.55

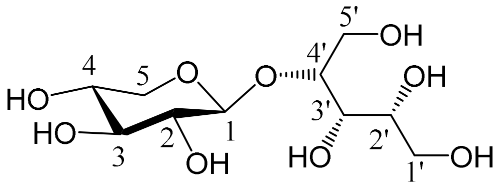

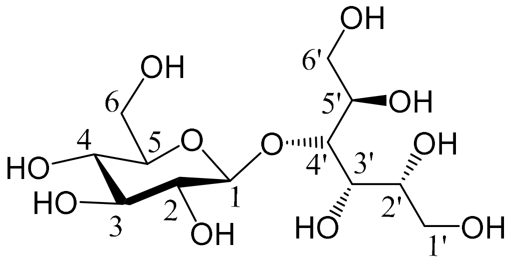

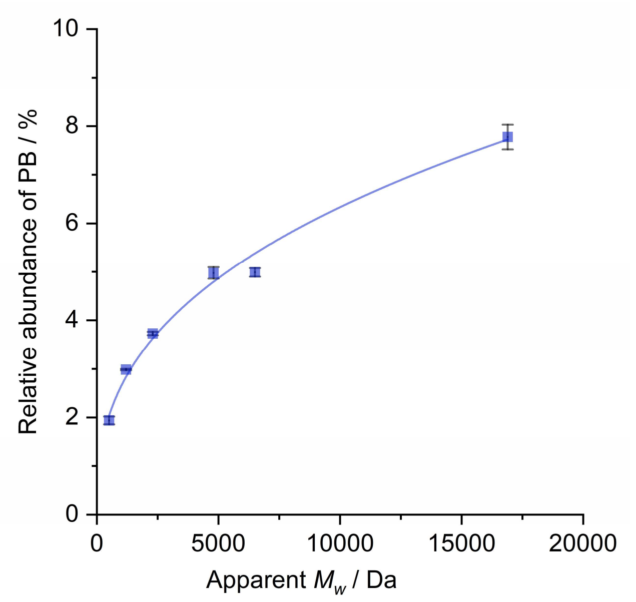

Figure S13. Correlation between the %PB units relative to combined G- and S-unit content and the apparent value of Mw of the fractions isolated by solvent fractionation of the RCF lignin oil.

References

[1] M. M. Abu-Omar, K. Barta, G. T. Beckham, J. S. Luterbacher, J. Ralph, R. Rinaldi, Y. Román-Leshkov, J. S. M. Samec, B. F. Sels, F. Wang, *Energy Environ. Sci.* **2021**, *14*, 262–292.

[2] C. M. Hansen, A. Björkman, *Holzforsch.* **1998**, *52*, 335–344.

[3] L. P. Novo, A. A. S. Curvelo, *Ind. Eng. Chem. Res.* **2019**, *58*, 14520–14527.

[4] W. C. O. Ribeiro, V. Lobosco, P. F. M. Martinez, *Bioresources* **2020**, *15*, 8577–8600.

[5] C. M. Hansen, *Hansen Solubility Parameters*, CRC Press, 2007.

[6] H. Kim, N. R. Vinueza, S. S. Kelley and S. Park, *Carbon Res. Convers.*, 2018, **1**, 238–244.
